# Resveratrol reduces ROS-induced ferroptosis by activating SIRT3 and compensating the GSH/GPX4 pathway

**DOI:** 10.1186/s10020-023-00730-6

**Published:** 2023-10-19

**Authors:** Xingjie Wang, Tianli Shen, Jie Lian, Kai Deng, Chao Qu, Enmeng Li, Gan Li, Yiwei Ren, Zijun Wang, Zhengdong Jiang, Xuejun Sun, Xuqi Li

**Affiliations:** 1 Department of General Surgery, The First Affiliated Hospital of Xi’an Jiaotong University, Xi’an, Shaanxi 710061 China; 2 Department of Pathology, The First Affiliated Hospital of Xi’an Jiaotong University, Xi’an, Shaanxi 710061 China; 3 Department of Talent Highland, The First Affiliated Hospital of Xi’an Jiaotong University, Xi’an, Shaanxi 710061 China

**Keywords:** Ischemia-reperfusion injury, Ferroptosis, SIRT3/FoxO3a pathway, Resveratrol, GSH/GPX4 pathway

## Abstract

**Background:**

Intestinal ischemia-reperfusion injury occurs in acute intestinal obstruction, intussusception, acute mesenteric artery embolism, and other diseases and can lead to local intestinal necrosis, distant organ involvement, or systemic reactions, with high morbidity and mortality. Ferroptosis plays a crucial role in intestinal ischemia-reperfusion injury, and inhibition of ferroptosis may provide new approaches for treating the disease. SIRT3 protects cells from oxidative stress and may be involved in the process of ferroptosis. We hypothesized that resveratrol, an agonist of SIRT3, could ameliorate intestinal ischemia-reperfusion injury by compensating the GSH/GPX4 pathway.

**Methods:**

Intestinal ischemia-reperfusion (I/R) and Caco-2 hypoxia-reoxygenation models were established. Transmission electron microscopy was used to assess mitochondrial function; the Chiu’s score was used to evaluate the degree of intestinal mucosal injury based on HE staining; and Western blot was used to detect the SIRT3/FoxO3a pathway, tight junction proteins and ferroptosis-related protein expression. *Sirt3*^-/-^ C57, sh*SIRT3*-Caco-2 cells and si*FoxO3a*-Caco-2 cells were established. C11-BODIPY was used to detect lipid peroxide in cells; FD4 and IFABP were used to detect intestinal permeability; MitoSOX was used to detect ROS levels; and MitoTracker and immunofluorescence colocalization were used to detect SIRT3 levels.

**Results:**

In the intestinal I/R model, I/R injury occurs mainly during the reperfusion period and leads to ferroptosis through the GSH/GPX4 pathway. Resveratrol could reduce ferroptosis and ameliorate I/R injury by activating SIRT3. In Sirt3^-/-^ mice, more intestinal mucosal cells underwent ferroptosis, I/R injury was more severe, and resveratrol lost the ability to ameliorate I/R injury. In addition, hypoxia-reoxygenation increased RSL3-induced ferroptosis sensitivity in Caco-2 cells in vitro. In the presence of sh*SIRT3* or RSL3 alone, resveratrol could ameliorate Caco-2 ferroptosis, but not RSL3-induced sh*SIRT3*-Caco-2 ferroptosis. Furthermore, resveratrol might activate the SIRT3/FoxO3a pathway, increase the expression of SOD2 and catalase, and inhibit ROS generation, thus reducing lipid peroxidation and ferroptosis.

**Conclusion:**

To date, this is the first study to show that resveratrol ameliorates intestinal ischemia-reperfusion injury by activating SIRT3 and reducing ferroptosis. Resveratrol can reduce intestinal ischemia-reperfusion injury by activating the SIRT3/FoxO3a pathway, increasing the expression of SOD2 and catalase, reducing ROS and LPO production, compensating for the GSH/GPX4 pathway and inhibiting ferroptosis.

**Graphical abstract:**

**Figure:**
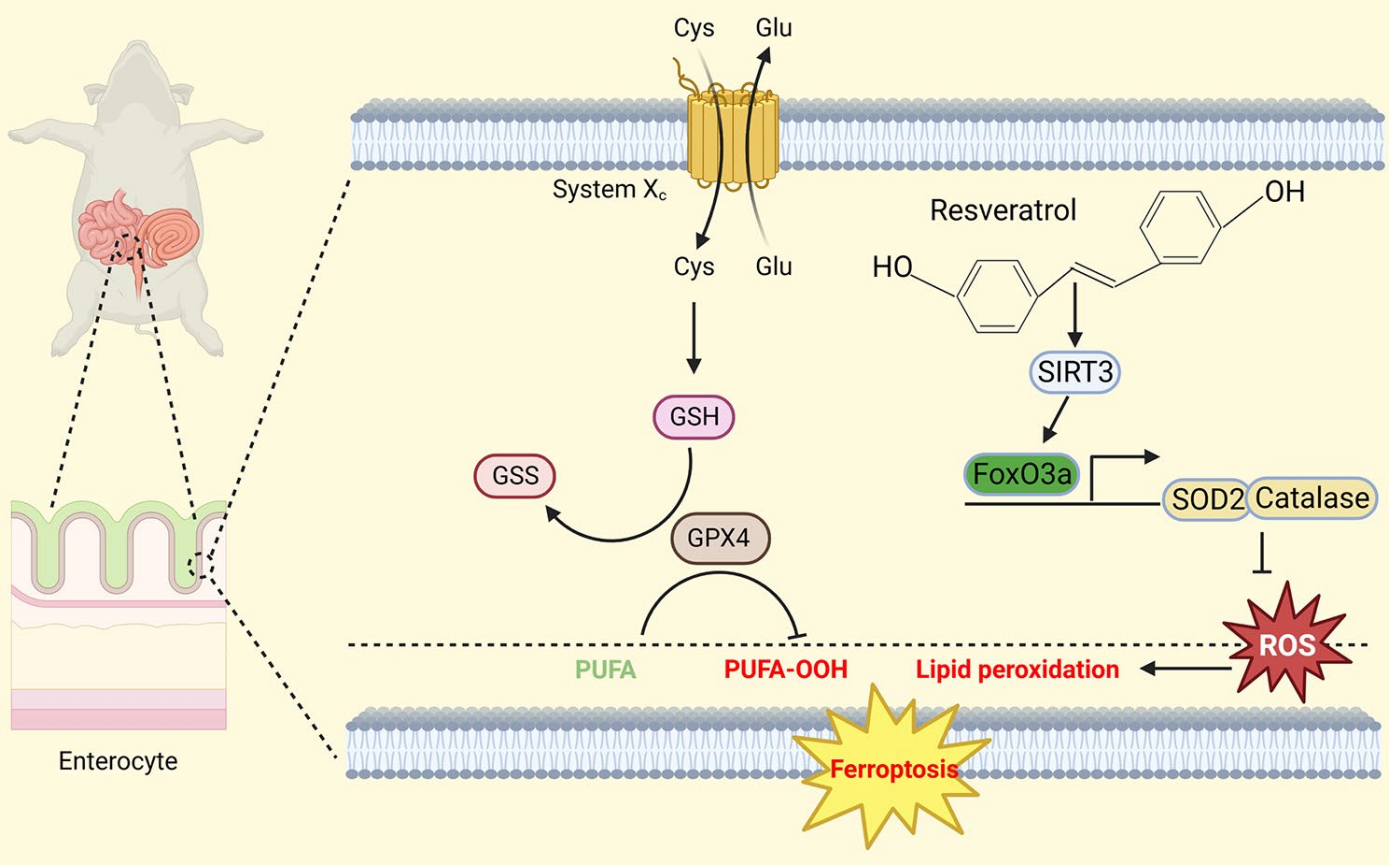
Resveratrol increases the expression of SOD2 and catalase, reduces the production of ROS and LPO, compensates for the GSH/GPX4 pathway and inhibits ferroptosis by activating the SIRT3/FoxO3a pathway

**Supplementary Information:**

The online version contains supplementary material available at 10.1186/s10020-023-00730-6.

## Introduction

Ischemia-reperfusion (I/R) injury is defined as a “second hit” injury occurring after the tissue or organ first suffers from an ischemic state and then blood flow is restored to the same tissue or organ. Traumatic shock, surgery, organ transplantation, burns, thrombus, etc., lead to I/R injury in organs such as the intestine, brain, liver, and heart (Eltzschig et al. 2011; Yan et al. 2020; Yellon et al. 2007). Intestinal I/R injury occurs in acute intestinal obstruction, intussusception, acute mesenteric artery embolism, etc., resulting in destruction of the intestinal barrier and changes in permeability, release of inflammatory mediators and bacterial toxins into the blood, and activation of reactive oxygen species (ROS), leading to distant organ involvement, such as acute lung injury or even multiple organ dysfunction syndrome (MODS), with a high incidence rate and a high mortality rate of up to 50% without specific treatment, resulting in a heavy economic burden to society (Li et al. 2019; Matsuo et al. 2013; Ucar et al. 2020).

Ferroptosis is an iron-dependent and lipid peroxidation (LPO)-induced programmed cell death that is different from apoptosis, pyroptosis, and necrosis. The hallmark feature of ferroptosis is mitochondrial membrane rupture under an electron microscope (Li et al. 2020a; Xie et al. 2016; Stockwell 2022). It is also manifested as a decrease in glutathione peroxidase 4 (GPX4), the core enzyme in the glutathione (GSH) antioxidant system (Chen et al. 2021a). Many studies have indicated that I/R injury induces ferroptosis (Wang et al. 2021a; Zhao et al. 2020, 2021; Li et al. 2020b, 2021). Ferroptosis is related to a variety of diseases, such as acute kidney injury (Wang et al. 2021b), cancer (Mou et al. 2019), cardiovascular disease (Wu et al. 2021a), neurodegenerative disease (Mahoney-Sánchez et al. 2021), and liver disease (Wu et al. 2021b).

When the accumulation of intracellular ROS overwhelms the effects of GSH and GPX4, lipid peroxidation occurs, leading to ferroptosis (Chen et al. 2021b). *SIRT3* is a class of highly conserved nicotinamide adenine dinucleotide (NAD^+^)-dependent deacetylases that are predominantly expressed in mitochondria and function in the regulation of oxidative stress, metabolism, inflammation, apoptosis and autophagy (Schwer et al. 2008). Shen et al. demonstrated that ROS production in the adherent tissues of *Sirt3*^*−/−*^ mice was obviously higher than that of *Sirt3*^*+/+*^ mice (Shen et al. 2022). *SIRT3* can reduce the expression of ROS by deacetylating superoxide dismutase 2 (SOD2) and catalase or by enhancing the transcription of *SOD2* and catalase through the SIRT3/FoxO3a pathway (Zhang et al. 2020; Fu et al. 2017; Bai et al. 2023). Therefore, we speculated that *SIRT3* functions in intestinal I/R injury by increasing the expression of FoxO3a.

Resveratrol (3,5,4′-trihydroxystilbene) is rich in grapes and products derived from grapes, such as red wine (Pastor et al. 2019). It was first extracted from *Veratrum grandiflorum O. Loes* and *Polygonum cuspidatum*, which are plants used in traditional Chinese medicine (Baur et al. 2006). Resveratrol has antioxidant, anti-inflammatory, and antitumor activities (Amri et al. 2012; Xia et al. 2017; de Sá Coutinho et al. 2018) and can also increase the expression of *SIRT3* to attenuate acute kidney injury, indicating that it is an activator of *SIRT3* (Zhang et al. 2020; Xu et al. 2016).

The aim of this study was to explore the possible mechanism by which resveratrol protects against intestinal I/R injury. To date, this is the first study to show that resveratrol ameliorates intestinal ischemia-reperfusion injury by activating SIRT3 and reducing ferroptosis. Here, we report that impaired expression of SIRT3 during I/R leads to a decrease in FoxO3a expression, which triggers the formation of excess ROS, leading to lipid peroxidation and ferroptosis. Furthermore, we demonstrate that resveratrol may be a novel therapeutic strategy for alleviating ROS, preventing ferroptosis, and attenuating intestinal I/R injury.

## Materials and methods

### Animals and drug treatment

Animal experiments were carried out with the approval of the Ethics Committee of the First Affiliated Hospital of Xi’an Jiaotong University (No. 2022 − 1240). Male C57BL/6 mice, 6–8 weeks old, 20 g ± 2 g, were purchased from the Laboratory Animal Center of Xi’an Jiaotong University. *Sirt3*^*−/−*^ mice were kindly supplied by the Jackson Laboratory. All mice were fed at 25 ℃ and 50-60% humidity with free access to water and food. 50 mg of resveratrol was added to 8.34 ml of drinking water and each mouse was gavaged with 100 ul of resveratrol for 2 weeks. Diluted resveratrol is for one-day use only and should be protected from light.

### Model of intestinal I/R

Mice were anesthetized with 5% isoflurane and disinfected with Aner iodine. A midline incision was made in the upper abdomen to expose and dissociate the mesenteric artery. Closure of the mesenteric artery was performed for 60 min with a small artery clip to simulate the ischemic process. The arterial clamp was released to simulate the reperfusion process. Three different reperfusion times, namely, 15 min, 30 min, and 60 min, were used. The method used to establish the I/R model is shown in Figure S1.

### Transmission electron microscopy

Intestinal tissue (1–2 mm^3^) was placed in an electron microscope fixative (G1102, Servicebio). The samples were dehydrated and embedded. Ultrathin tissues were stained and observed with an HT7700 transmission electron microscope at 80 kV.

### Cell culture and an hypoxia-reoxygenation (H/R) model

Caco-2 colorectal adenocarcinoma (CL-0050, ProCell) cells were cultured in minimum essential medium (MEM, Gibco) with 20% fetal bovine serum (FBS, 10,091,148, Gibco) in a 5% CO_2_ incubator at 37 °C. Caco-2 cells were incubated in a microaerophilic system (Thermo Fisher) containing 1% O_2,_ 5% CO2, and 94% N_2_ for 12 h to induce an H/R model.

### Construction of stable sh*SIRT3* and si*FoxO3a* cell lines

Caco-2 cells were infected with sh*SIRT3* lentiviruses (GenePharma). Seventy-two hours after infection, sh*SIRT3*-Caco-2 cells were selected with 3 µg/mL puromycin (A1113803, Gibco) for one week. Caco-2 cells were infected with si*FoxO3a* plasmid (Tsingke), and RNA and protein were extracted. sh*SIRT3* (forward: 5′-ccggGTGGGTGCTTCAAGTGTTGTTctcgagAACAACACTTGAAGCACCCACtttttg-3′ and reverse: 5′-aattcaaaaaGTGGGTGCTTCAAGTGTTGTTctcgagAACAACACTTGAAGCACCCAC-3′). si*FoxO3a*-1 (forward: 5′-GGAACGUGAUGCUUCGCAA-3′ and reverse: 5′- UUGCGAAGCAUCACGUUCC-3′), si*FoxO3a*-2 (forward: 5′-GCUCUUGGUGGAUCAUCAA-3′ and reverse: 5′- UUGAUGAUCCACCAAGAGC-3′).

### Drug treatment and CCK8 assay

RSL3 (IR1120, Solarbio) was used to induce ferroptosis in Caco-2 cells at concentrations of 2, 4, 8, 12, 16, 20, 24 and 28 µM for 24 h. Resveratrol (GC14553, GlpBio) was dissolved in DMSO at a concentration of 10 mM. Caco-2 cells were seeded in 96-well culture plates (500 cells per well) and treated with resveratrol at concentrations of 2.5, 5, 10, 20 and 40 µΜ for 24 h, 48 h, or 72 h. Ten microliters of CCK8 reagent (C0005, TargetMol) and 90 µl of MEM were added to each well at 37 °C for 2 h. Before the test, 96-well plates were placed on a shaker for 5 min. Finally, the cell viability was quantified using a microplate reader at 450 nm. All experiments were performed in triplicate.

### LIVE/DEAD assay

As in the CCK8 assay, 100 µl of LIVE/DEAD (L3224, Thermo Fisher Scientific) working solution was added to the wells. After 20 min of incubation in dark conditions at room temperature, the signal was visualized with a fluorescence microscope (DM IL LED, Leica) at 200X.

### Western blot

Proteins were obtained from Caco-2 cells and intestinal tissues, resolved by electrophoresis, transferred to nitrocellulose filter membranes (HATF00010, Sigma), blocked with 5% skim milk in TBST, incubated with primary antibodies overnight at 4 °C and incubated with a secondary antibody for 1 h at room temperature. Electrogenerated chemiluminescence (ECL, P10300, NcmBiotech) produced the corresponding protein color. GAPDH was used as a control for whole homogenates. The results were analyzed for gray values using Fiji software. The primary antibodies used were anti-GAPDH (5174, Cell Signaling Technology), anti-Occludin (91,131, Cell Signaling Technology), anti-SIRT3 (5490, Cell Signaling Technology), anti-GPX4 (59,735, Cell Signaling Technology), anti-FTH1 (4393, Cell Signaling Technology), anti-ACSL4 (ab155282, Abcam), anti-ZO-1 (13,663, Cell Signaling Technology), anti-SIRT4(69,786, Cell Signaling Technology), anti-SIRT5(8782, Cell Signaling Technology), anti-SOD2 (24127-1-AP, Proteintech), anti-catalase (24127-1-AP, Proteintech) and anti-FoxO3a (10894-1-AP, Proteintech), and secondary antibody (7074, Cell Signaling Technology).

### Measurement of inflammatory factor levels

Blood samples were taken from the inferior vena cava of anesthetized mice and stored in a coagulation tube. Centrifugation was carried out at 1500 × g for 10 min to separate the serum from the cells. The serum was then effectively collected. An ELISA kit was used to test interleukin-6 levels (IL6, ab100712, Abcam), tumor necrosis factor α levels (TNF-α, ab208348, Abcam), and lactate dehydrogenase levels (LDH, ab102526, Abcam) according to the manufacturer’s instructions. Before measuring LDH, the serum was diluted 1/10. The levels of IL6, TNF-α and LDH were measured with a microplate reader at 450 nm.

### Intestinal permeability assay

According to the studies of Fox et al. (Fox et al. 2012) and Liu et al. (Liu et al. 2020), fluorescein isothiocyanate dextran 4 (FD4, 60,842, Sigma) was dissolved in normal saline at a dose of 0.5 mg/g. Mice were fasted for 8 h and gavaged with 0.5 ml of FD4 solution 3 h before ischemia. Serum was collected as described above. The concentration of FD4 was read with a microplate reader using an excitation wavelength of 490 nm and an emission wavelength of 520 nm. An intestinal fatty acid binding protein (IFABP) kit (abx153976, abbexa) was used to test intestinal permeability according to the instructions, and the absorbance at 450 nm was measured.

### Iron, MDA, and glutathione assays

The total iron, Fe^2+^ and Fe^3+^ levels were assessed with an iron test kit (ab83366, Abcam) with a microplate reader at OD 593 nm. Malondialdehyde (MDA) was assessed with a Lipid Peroxidation Assay kit (ab118970, Abcam) at OD 532 nm. Glutathione, GSH, GSSG and GSH/GSSG levels were assessed using the GSH + GSSG/GSH Assay Kit (ab239709, Abcam) at OD 412 nm. Tissue samples and cell samples were prepared according to the manufacturer’s protocols.

### Lipid peroxidation (LPO) assay

The intensely fluorescent BODIPY is an effective tracer of lipid trafficking (Bai et al. 1997). The C11-BODIPY 581/591 kit (D3861, Thermo Fisher) was used to test the LPO concentration in cells following the manufacturer’s instructions. Briefly, after cell treatment, 5 µM reagent was added to 96-well plates, and the cells were incubated for 30 min at 37 °C, washed with PBS three times, stained with 5 µg/ml Hoechst (33,342, Thermo Fisher) for 30 min at 37 °C, photographed under a fluorescence microscope (DM IL LED, Leica) and analyzed with Fiji software.

### Hematoxylin and eosin (HE) staining

After reperfusion, intestinal samples were collected and fixed in 4% PFA (BL539A, Biosharp, China) at RT for 24 h. After dehydration, 5 μm sections were stained with HE. The staining was scored by three pathologists blinded to this research according to the methods of Chiu et al. (Chiu et al. 1970) and Hacioglu et al. (Hacioglu et al. 2005). A morphometric study was conducted using a Phmias microscope (MC-D310U/C).

### Real-time polymerase chain reaction (PCR) analysis

Total RNA was obtained from Caco-2 cells treated with or without resveratrol using TRIzol reagent (15596018, Thermo Fisher). Complementary DNA was synthesized with All-In-One 5X RT MasterMix (592, abm). Quantitative PCR was performed with a SYBR Green assay (25741, Thermo Fisher). As the gene of interest, RT-PCR was run for *SIRT3, SIRT4, SIRT5*, and *FoxO3a*. GAPDH served as a reference gene. The primer sequences were as follows: *SIRT3*: (forward: 5’- CCCTGGAAACTACAAGCCCAAC-3’ and reverse: 5’- GCAGAGGCAAAGGTTCCATGAG–3’); *SIRT4*: (forward: 5′-CAGCAAGTCCTCCTCTGGAC-3′ and reverse: 5′-CCAGCCTACGAAGTTTCTCG-3′); *SIRT5*: (forward: 5′-TGGCTCGGCCAAGTTCAAGTATG-3′ and reverse: 5′-AAGGTCGGAACACCACTTTCTGC-3′); *FoxO3a*: (forward: 5′-CTTGATGTCTCAGGCCAGCA-3′ and reverse: 5′-CAAGTCGCTGGGGAACTTCT-3′); and GAPDH: (forward: 5’- CCACCCATGGCAAATTCCATGGCA-3’, and reverse: 5’- TCTAGACGGCAGGTCAGGTCCACC-3’).

### ROS measurement

The level of ROS in the cells was detected with a MitoSOX Red kit (M36008, Thermo Fisher). After treatment, the cells were stained with 5 µM MitoSOX Red in HBSS/Ca/Mg solution, incubated for 10 min at 37 °C and washed with prewarmed HBSS/Ca/Mg solution three times. Next, the cells were stained with 5 µg/ml Hoechst for 30 min at 37 °C and washed with warm HBSS/Ca/Mg. The mean fluorescence intensity (MFI) was imaged by fluorescence microscopy (DM IL LED, Leica) and analyzed with Fiji software.

### MitoTracker Red and immunofluorescence (IF) staining

After treatment, the cells were stained with the MitoTracker Red kit (M7512, Invitrogen, Thermo Fisher) in prewarmed HBSS/Ca/Mg for 30 min at 37 °C and washed with warm HBSS/Ca/Mg three times. The cells were incubated with the primary antibody for 2 h at room temperature, washed with prewarmed HBSS/Ca/Mg three times and incubated with secondary antibody (11,008, Thermo Fisher) and DAPI (36,935, Thermo Fisher) at the same time for 1 h at room temperature. After the cells were washed with prewarmed HBSS/Ca/Mg, the MFI was observed by fluorescence microscopy (DM IL LED, Leica) and analyzed with Fiji software. The primary antibodies used were anti-SIRT3 (5940, Cell Signaling Technology) and anti-SOD2 (13,534, Abcam).

### Statistical analysis

GraphPad Prism 9.0 was used to analyze the data. GraphPad Prism 9.0 was used to analyze the data. In vitro studies, data are mean ± SD of three independent experiments in triplicates (n = 3). In vivo studies (including samples from mice), data are expressed as the means ± SD (n = 8), all experiments were repeated in triplicate. Normality was tested by the Shapiro-Wilk test. Measurement data were analyzed by Student’s t test or analysis of variance (ANOVA), and ranked data were analyzed by the Kruskal-Wallis H test. *P* < 0.05 was considered significant.

## Results

### I/R injury and ferroptosis occur in a mouse model of intestinal I/R

**Fig. 1:**
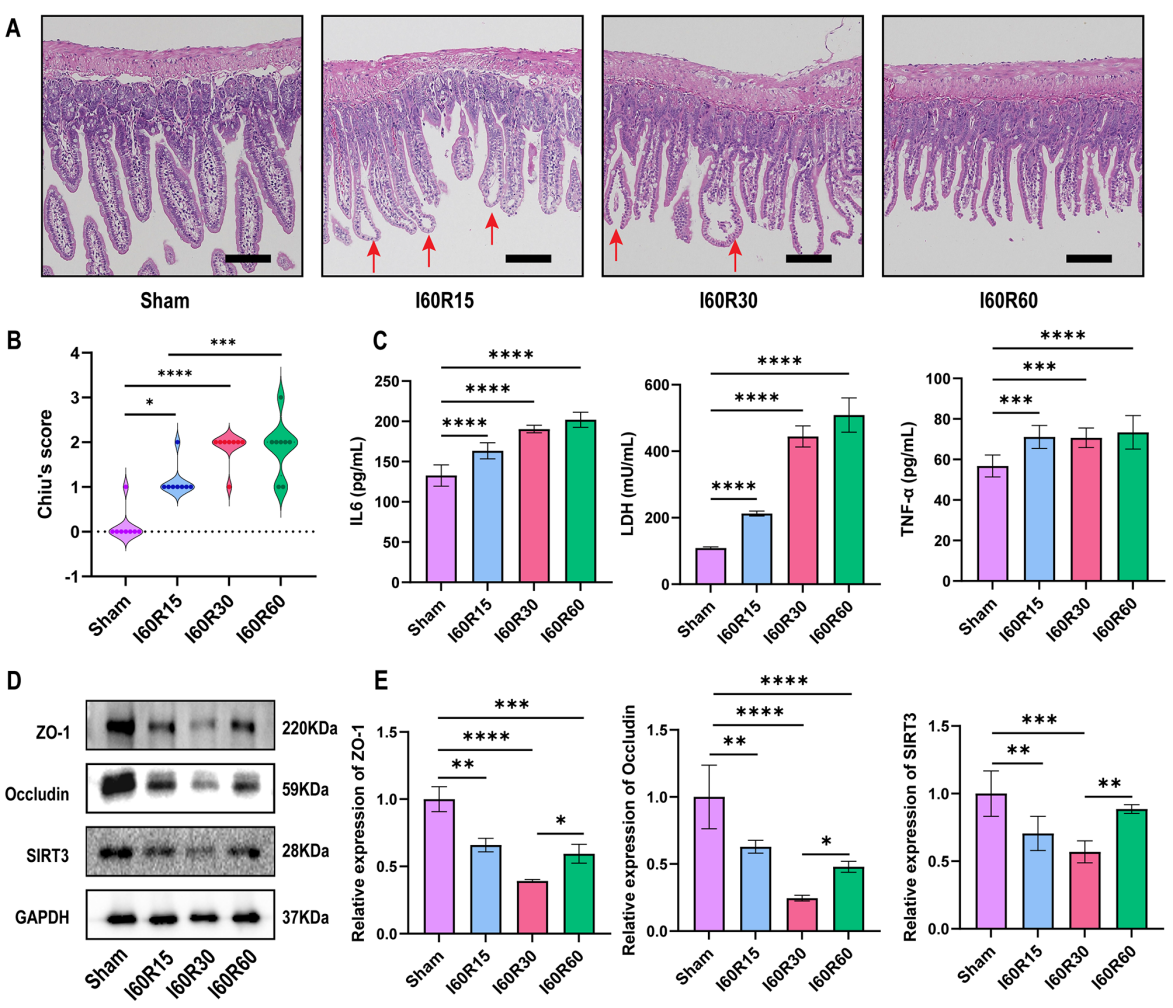
**I/R injury occurs in a mouse model of intestinal I/R**. (**a**) Representative HE staining images of intestinal mucosa after 15 min, 30 and 60 min of reperfusion (n = 8). Scale bars: 200 μm. (**b**) Chiu’s score of intestinal tissue injury after different reperfusion times (n = 8). (**c**) Quantitation of IL6, LDH, and TNF-α in serum after 15 min, 30 and 60 min of reperfusion (n = 8). (**d**) Representative Western blots of the TJ proteins ZO-1, Occludin, and SIRT3 after 15 min, 30 and 60 min of reperfusion (n = 8). (**e**) Quantitation of TJ proteins expressed after different reperfusion times (n = 8). The red arrow represents the Gruen-Hagen space. Data are presented as the mean ± SD. All experiments were repeated in triplicate. * *P* < 0.05, **P < 0.01, ***P < 0.001, ****P < 0.0001. Kruskal-Wallis H test (b), one-way ANOVA (c, e)

We generated an intestinal I/R model and studied intestinal injury at different reperfusion times when the ischemia time was fixed at 60 min. Figure 1a-b shows that reperfusion injury had already occurred at 15 min of reperfusion, and the Gruen-Hagen space (marked with a red arrow) was developing at the tip of the villus. Reperfusion injury was the most serious at 30 and 60 min of reperfusion, the intestinal mucosa was damaged and broken, and intestinal mucosa continuity was deteriorated. Due to reperfusion injury, serum expression levels of LDH and the inflammatory factors IL6 and TNF-α increased with time, and did not decrease at 60 min of reperfusion (Fig. 1c). Tight junctions (TJs) maintain the permeability and physical barrier of the intestinal mucosa (Bhat et al. 2018). The difference in ZO-1 and Occludin expression levels at different reperfusion times was assessed by Western blot. Consistent with the HE staining, the levels of ZO-1 Occludin and SIRT3 were the lowest at 30 min of reperfusion, and increased at 60 min of reperfusion compared with 30 min of reperfusion (Fig. 1d-e).

**Fig. 2:**
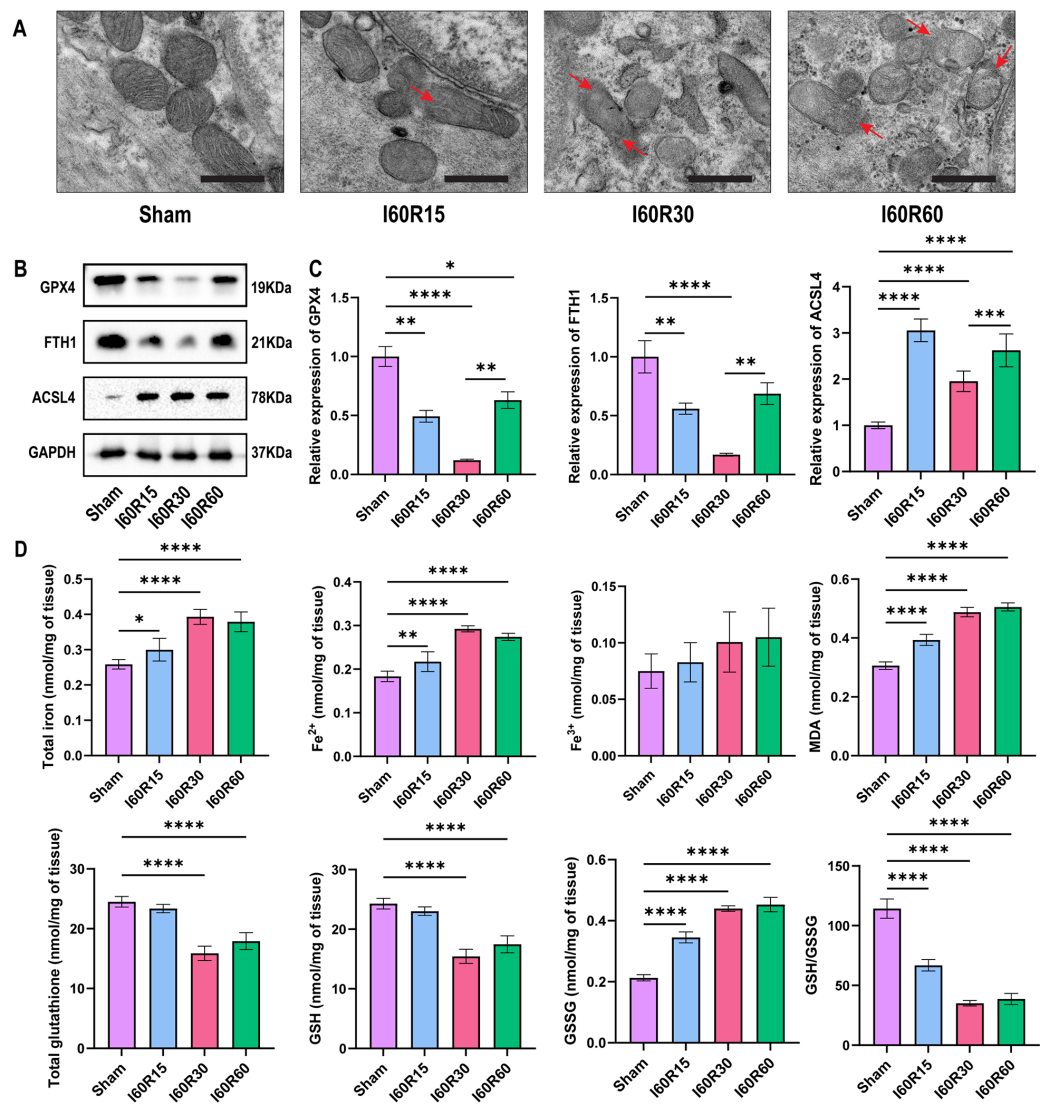
**Ferroptosis occurs in a mouse model of intestinal I/R**. (**a**) Representative transmission electron microscopy images showing mitochondrial structure and morphology (n = 8). Scale bars: 500 nm. (**b**) Representative Western blots of the ferroptosis-associated proteins GPX4, FTH1 and ACSL4 after 15 min, 30 and 60 min of reperfusion (n = 8). (**c**) Quantitation of ferroptosis-associated proteins expressed after different reperfusion times (n = 8). (**d**) Total iron, Fe^2+^, Fe^3+^, MDA, total glutathione, GSH, GSSG, and GSH/GSSG levels were evaluated in reperfusion tissues (n = 8). The red arrows represent ruptured mitochondrial membranes. Data are presented as the mean ± SD. All experiments were repeated in triplicate. *P < 0.05, **P < 0.01, ***P < 0.001, ****P < 0.0001. One-way ANOVA (c, d)

We also observed that ferroptosis occurred during I/R and was correlated with reperfusion time. The morphology of mitochondria was observed by TEM to evaluate the degree of ferroptosis. After 15 min of reperfusion, the outer mitochondrial membrane (OMM) ruptured (marked with red arrows) and worsened after 30 and 60 min of reperfusion (Fig. 2a). The Western blot results showed that the GPX4 and FTH1 expression levels were the lowest and that the ACSL4 expression level was the highest after 30 min of reperfusion (Fig. 2b-c). After 30 min of reperfusion, total iron and Fe^2+^ levels in the tissue were the highest, the LPO product MDA also accumulated with prolonged reperfusion time, the level of reduced GSH decreased, and the level of oxidized GSSG increased (Fig. 2d). In subsequent in vitro experiments, ischemia for 60 min and reperfusion for 30 min (I60R30) were used to induce intestinal I/R injury. These results suggest that reperfusion injury and ferroptosis were most severe after 30 min of reperfusion.

### H/R increases the sensitivity of RSL3 to induce ferroptosis in Caco-2 cells

**Fig. 3:**
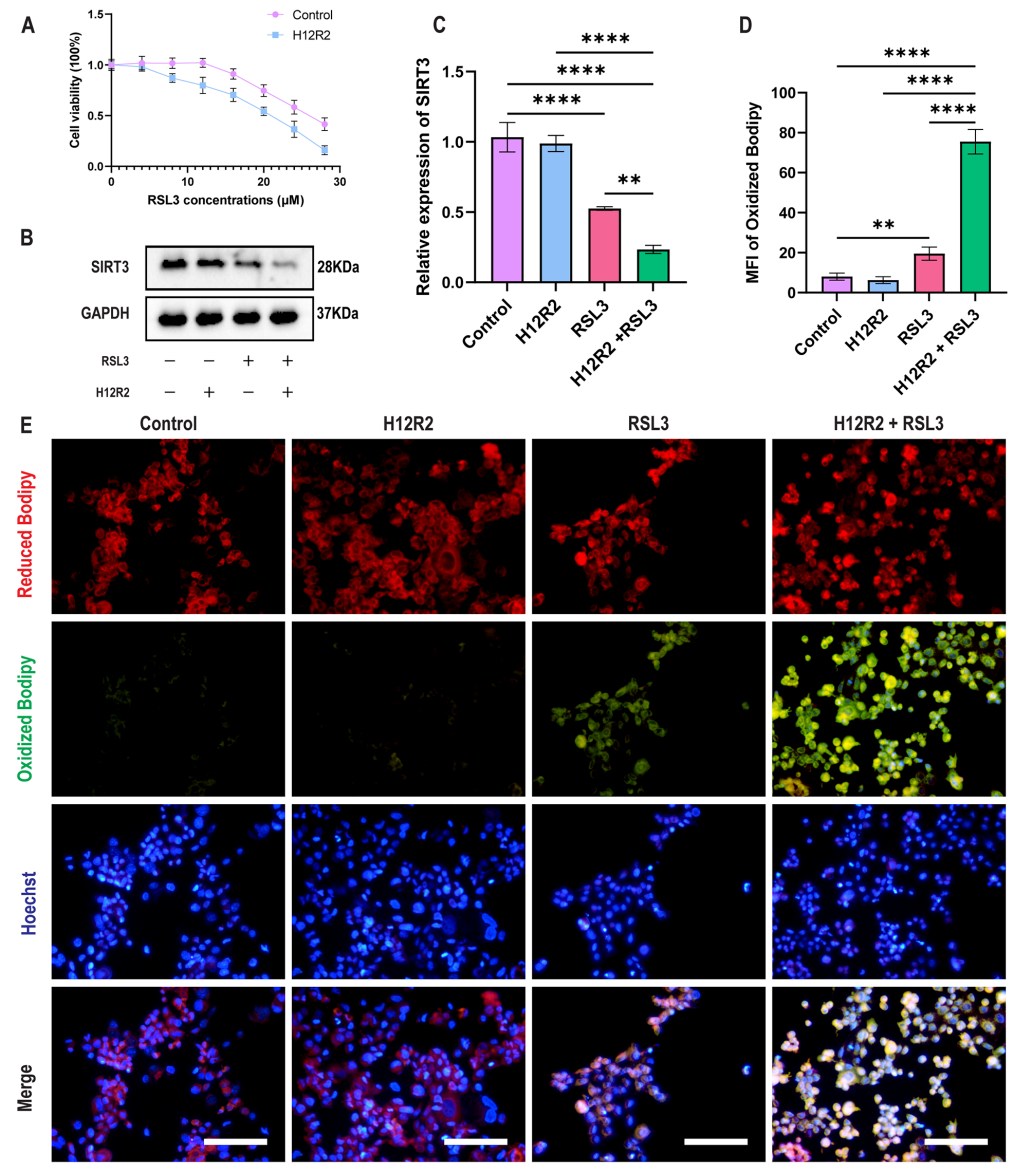
** H/R increases the sensitivity of RSL3 to induce ferroptosis in Caco-2 cells**. (**a**) Viability of cells treated with different concentrations of RSL3 for 24 h with or without H/R (n = 3). (**b**) Representative Western blots of SIRT3 treated with or without H/R and/or RSL3 (n = 3). (**c**) Quantitation of SIRT3 treated with or without H/R and/or RSL3 (n = 3). (**d**) Quantification of the MFI of the IF image (n = 3). (**e**) Representative IF image indicating LPO (green fluorescence) in cells treated with or without H/R and/or RSL3 (n = 3). Scale bars: 200 μm. Data are presented as the mean ± SD of three independent experiments in triplicates. **P < 0.01, ****P < 0.0001. Two-way ANOVA (c, d)

To verify whether H/R affects ferroptosis in vitro, according to the study of Li et al. (Li et al. 2019), we established an H/R model with a fixed hypoxia time of 12 h and reoxygenation time of 2 h. Caco-2 cells were treated with different concentrations of RSL3 with or without H/R. We found that 16 µM RSL3 decreased the activity of Caco-2 cells without H/R, while 8 µM RSL3 caused a decrease in Caco-2 cell viability with H/R (Fig. 3a). Compared with RSL3alone, RSL3 combined with H/R increased the total iron, Fe^2+^, Fe^3+^, MDA and GSSG levels, while the total glutathione and GSH levels and the GSH/GSSG ratio decreased, suggesting that in the presence of H/R, cell ferroptosis was higher (Figure S2a). We also found that the SIRT3 expression level in Caco-2 cells was the lowest in the presence of H/R and RSL3 (Fig. 3b-c). C11-BODIPY 581/591 is a fluorescent probe for lipid peroxidation. In the RSL3 combined with H/R group, the MFI of oxidized BODIPY increased (Fig. 3d-e). These results suggest that H/R did not directly induce Caco-2 ferroptosis but increased the sensitivity of RSL3-induced ferroptosis. In the next experiment, 16 µM RSL3 and H/R were used to induce ferroptosis in Caco-2 cells to mimic the process of I/R in vivo.

### *Sirt3*^−/−^ increases intestinal I/R injury and ferroptosis in an I/R model

**Fig. 4:**
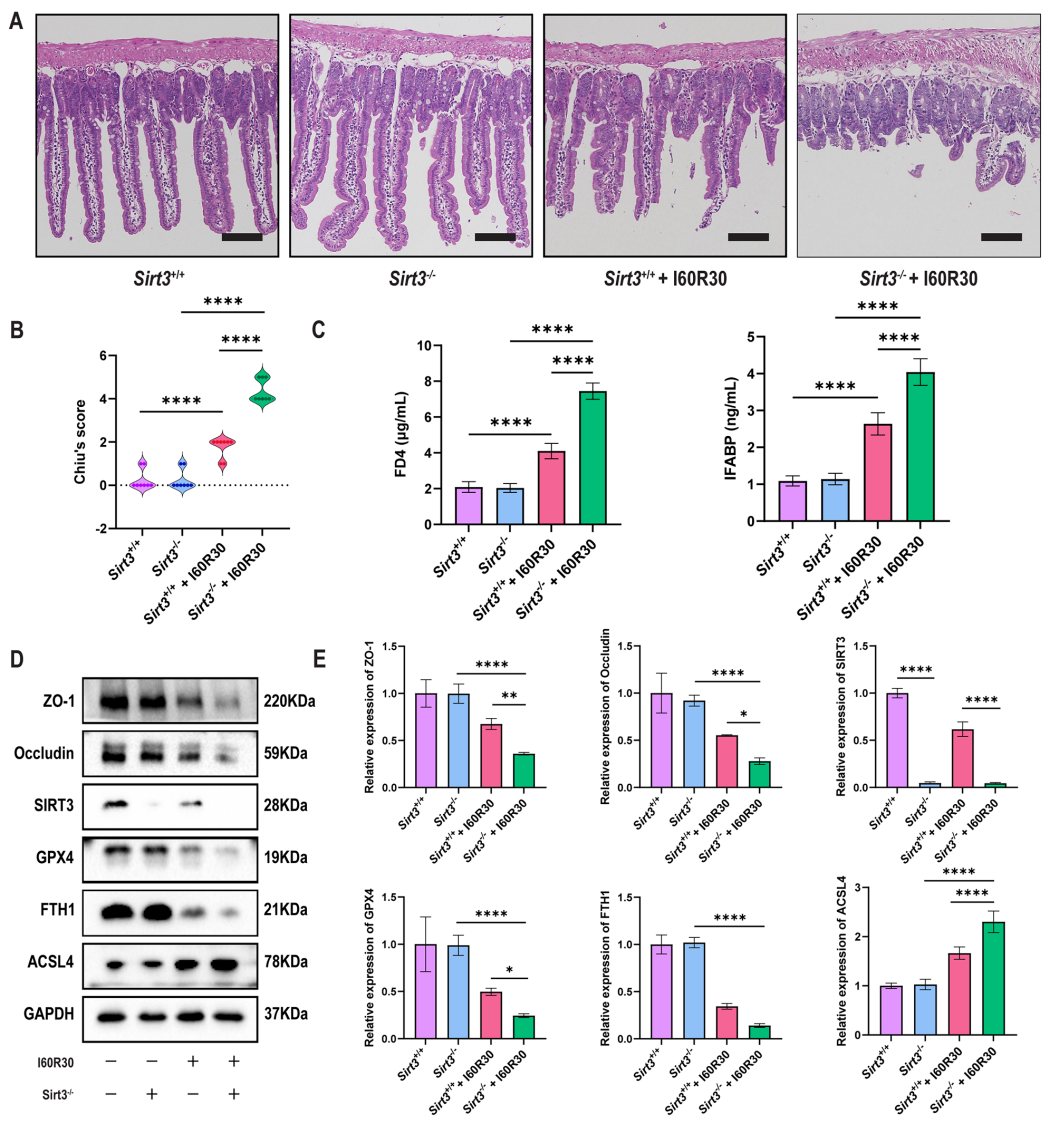
***Sirt3***^**−/−**^**increases intestinal I/R injury and ferroptosis in a mouse I/R model**. (**a**) Representative HE staining images of the intestinal mucosa of *Sirt3*^+/+^ and *Sirt3*^−/−^ mice after I/R or without I/R (n = 8). Scale bars: 200 μm. (**b**). Chiu’s score of intestinal tissue injury in *Sirt3*^+/+^ and *Sirt3*^−/−^ mice after I/R or without I/R (n = 8). (**c**) Quantification of FD4 and IFABP levels in the serum of *Sirt3*^+/+^ and *Sirt3*^−/−^ mice after I/R or without I/R (n = 8). (**d**) Representative Western blots of ZO-1, Occludin, SIRT3, GPX4, FTH1, and ACSL4 levels from intestinal tissue of *Sirt3*^+/+^ and *Sirt3*^−/−^ mice after I/R or without I/R (n = 8). (**e**) Quantification of these proteins from intestinal tissues of *Sirt3*^+/+^ and *Sirt3*^−/−^ mice after I/R or without I/R (n = 8). Data are presented as the mean ± SD. All experiments were repeated in triplicate. *P < 0.05, **P < 0.01, ****P < 0.0001. Kruskal-Wallis H test (b), Two-way ANOVA (c, e)

*SIRT3* is a mitochondrial sirtuin and exhibits NAD^+^-dependent deacetylase activity, which plays an important role in many diseases (Zhang et al. 2020). SIRT3 expression was decreased after I/R in intestinal tissues (Fig. 1c-d). Compared to *Sirt3*^+/+^ mice, *Sirt3*^−/−^ mice had more severe intestinal mucosal damage and higher Chiu’s score after I/R, but the intestinal mucosa was not damaged in either *Sirt3*^+/+^ or *Sirt3*^−/−^ mice without I/R (Fig. 4a-b). IL-6, LDH and TNF-α levels were increased in the serum of *Sirt3*^*−/−*^ mice, suggesting injury and local inflammation of the intestinal mucosa (Figure S3a), and serum FD-4 and IFABP levels were increased, which suggested increased intestinal permeability after 60 min of intestinal ischemia and 30 min of reperfusion (Fig. 4c). Similar to the HE results, the Western blot results suggested that the levels of ZO-1 and Occludin, GPX4 and FTH1 decreased and that of ACSL4 increased in *Sirt3*^−/−^ mice after 60 min of intestinal ischemia and 30 min of reperfusion (Fig. 4d-e).

### *SIRT3* knockdown increases sensitivity to RSL3-induced ferroptosis

**Fig. 5:**
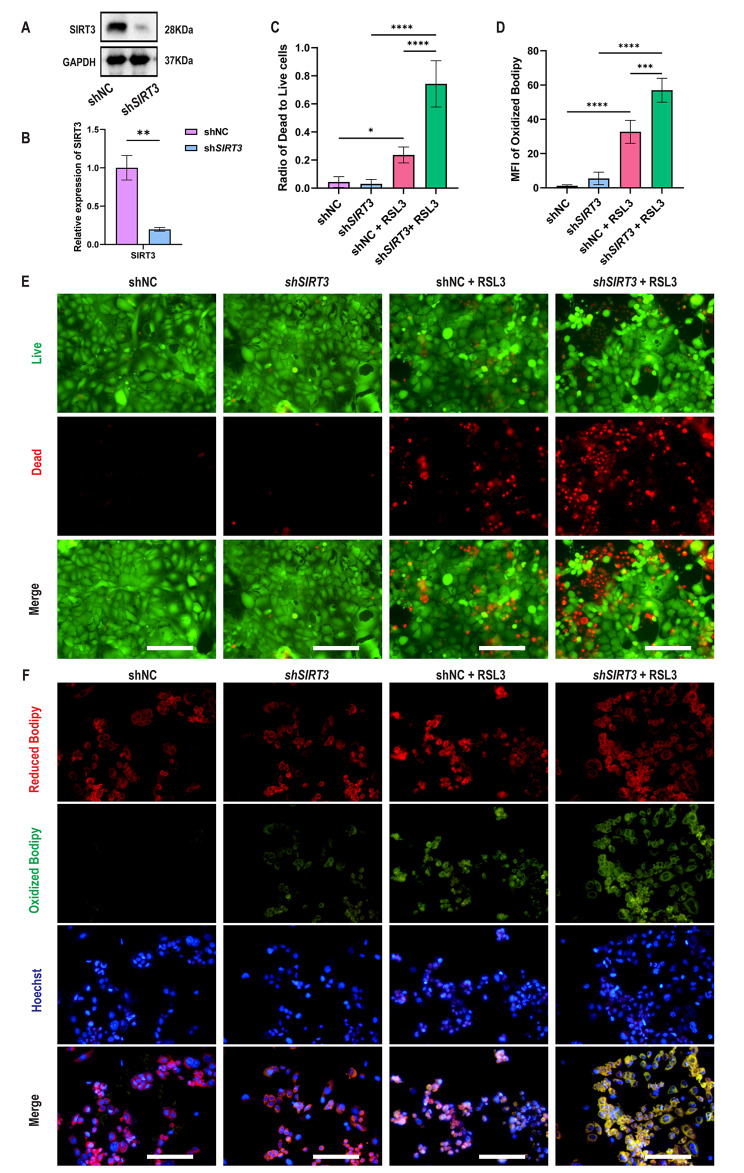
***SIRT3*knockdown increases sensitivity to RSL3-induced ferroptosis**. (**a**) Representative Western blots of SIRT3 expressed in shNC and sh*SIRT3*-Caco-2 cells (n = 3). (**b**) Quantification of SIRT3 expression in shNC and sh*SIRT3*-Caco-2 cells (n = 3). (**c**) Quantitation of cell viability in shNC and sh*SIRT3*-Caco-2 cells treated with or without RSL3 (n = 3). (**d**) Representative LIVE/DEAD staining images showing cell viability in shNC and sh*SIRT3*-Caco-2 cells treated with or without RSL3 (n = 3). Scale bars: 200 μm. (**d**) Quantification of oxidized BODIPY in shNC and sh*SIRT3*-Caco-2 cells treated with or without RSL3 (n = 3). (**f**) Representative fluorescence images showing the levels of reduced BODIPY, oxidized BODIPY and Hoechst in shNC and sh*SIRT3* Caco-2 cells treated with or without RSL3 (n = 3). Scale bars: 200 μm. Data are presented as the mean ± SD of three independent experiments in triplicates. *P < 0.05, ***P < 0.001, ****P < 0.0001. Student’s t test (b), two-way ANOVA (c, d)

Stable shNC and sh*SIRT3* Caco-2 cells were successfully established in vitro (Fig. 5a-b). Furthermore, to evaluate the effect of *SIRT3* knockdown on ferroptosis, LIVE/DEAD staining was performed. The results showed that the ratio of dead cells (red fluorescence) to live cells (green fluorescence) was higher in sh*SIRT3*-Caco-2 cells treated with RSL3 than in shNC-Caco-2 cells. The ratio of dead cells to living cells did not differ between sh*SIRT3*-Caco-2 cells and shNC-Caco-2 cells, which indicated that the elimination of *SIRT3* did not affect cell viability but increased susceptibility to RSL3-induced cell death (Fig. 5c and e). After sh*SIRT3*-Caco-2 cells were treated with RSL3, total iron, Fe^2+^, Fe^3+^, MDA and GSSG levels increased, while total glutathione and GSH levels and the GSH/GSSG ratio decreased (Figure S4a). The C11-BODIPY 581/591 kit is a lipid peroxide sensor, and unoxidized BODIPY appears red and oxidized BODIPY appears green. The MFI of oxidized BODIPY (green fluorescence) increased, which indicated an increase in LPO in sh*SIRT3*-Caco-2 cells treated with RSL3 (Fig. 5d and f).

### Resveratrol ameliorates intestinal I/R injury in an I/R model

**Fig. 6:**
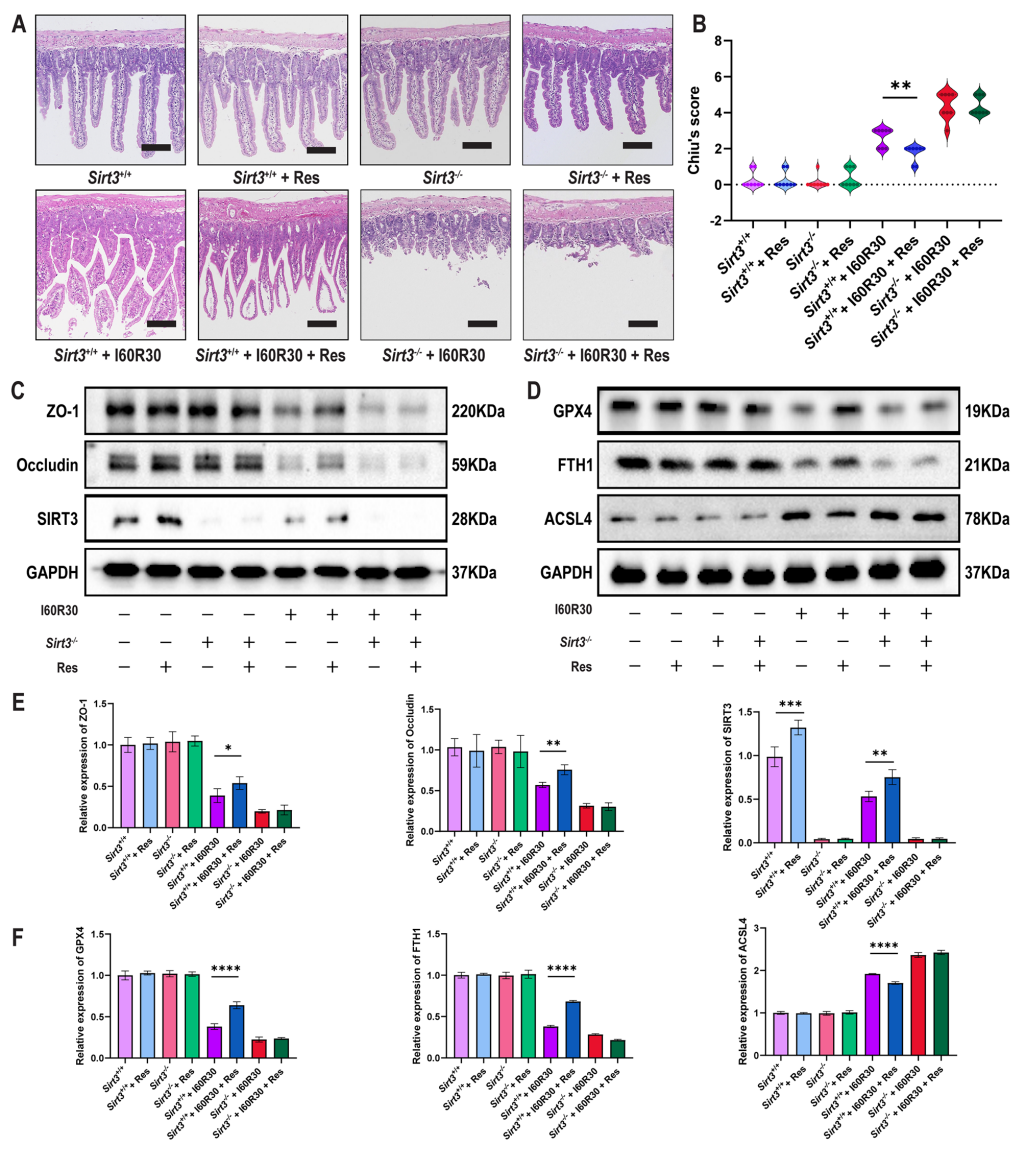
**Resveratrol ameliorates intestinal I/R injury in a mouse I/R model**. (**a**) Representative HE staining images of intestinal mucosa after drinking resveratrol (30 mg/L) for 2 weeks (n = 8). Scale bars: 200 μm. (**b**) Chiu’s score of intestinal tissue injury in *Sirt3*^+/+^ and *Sirt3*^*−/−*^ mice treated with resveratrol and/or I/R (n = 8). (**c**) Representative Western blots of ZO-1 and Occludin in *Sirt3*^+/+^ and *Sirt3*^−/−^ mice treated with resveratrol and/or I/R (n = 8). (**d**) Representative Western blots of GPX4, FTH1 and ACSL4 levels in *Sirt3*^+/+^ and *Sirt3*^−/−^ mice treated with resveratrol and/or I/R (n = 8). (**e**) Quantitation of ZO-1 and Occludin in *Sirt3*^+/+^ and *Sirt3*^−/−^ mice treated with resveratrol and/or I/R (n = 8). (**f**) Quantitation of GPX4, FTH1 and ACSL4 levels in *Sirt3*^+/+^ and *Sirt3*^−/−^ mice treated with resveratrol and/or I/R (n = 8). Data are presented as the mean ± SD. All experiments were repeated in triplicate. *P < 0.05, **P < 0.01, ***P < 0.001. Kruskal-Wallis H test (b), two-way ANOVA (e, f)

Resveratrol, a kind of *SIRT3* activator, is a chemical found in red grapes and products made from red grapes, such as wine and juice (Zhang et al. 2020; Amri et al. 2012). The intake of a certain amount of resveratrol did not cause damage to intestinal tissue. Resveratrol ameliorated I/R injury in *Sirt3*^+/+^ mice but not in *Sirt3*^−/−^ mice (Fig. 6a-b). The Western blot results suggested that resveratrol also increased the expression of SIRT3, the tight junction proteins ZO-1 and Occludin and the ferroptosis-associated proteins GPX4 and FTH1in *Sirt3*^+/+^ mice due to I/R injury but not in *Sirt3*^−/−^ mice (Fig. 6c-f). Consistent with the results of HE and Western blot, resveratrol reduced the high expression levels of IL6, TNF-α, and LDH in serum after intestinal I/R injury in *Sirt3*^+/+^ mice but not in *Sirt3*^−/−^ mice (Figure S5a).

### Resveratrol attenuates RSL3-induced ferroptosis in a manner dependent on SIRT3 activation

**Fig. 7:**
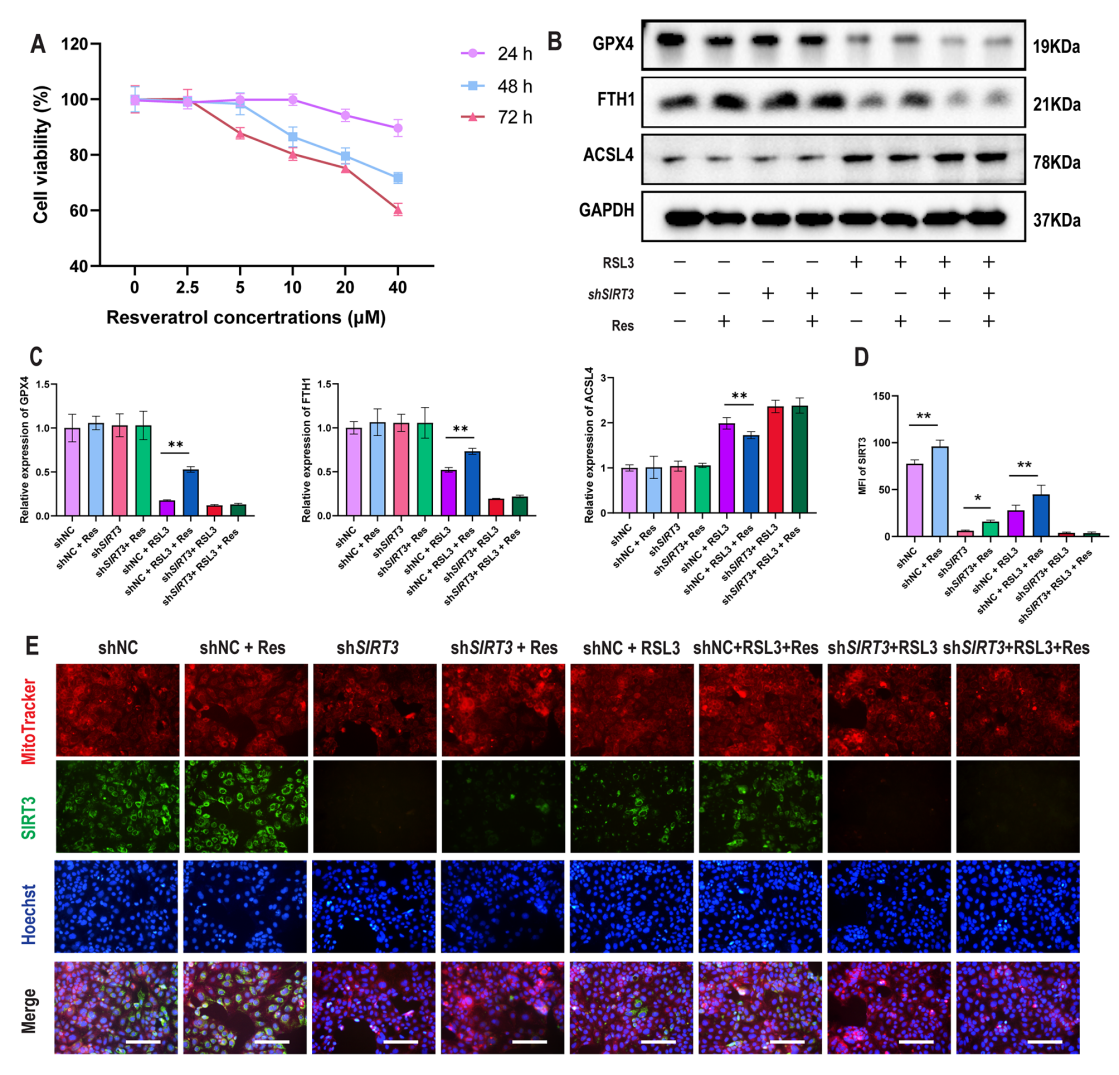
**Resveratrol attenuates RSL3-induced ferroptosis dependent on*SIRT3*activation**. (**a**) Viability of Caco-2 cells treated with resveratrol at concentrations of 0, 2.5, 5, 10, 20, and 40 µM for 24 h, 48 and 72 h (n = 3). (**b**) Representative Western blots of GPX4, FTH1 and ACSL4 levels in shNC-Caco-2 cells and sh*SIRT3*-Caco-2 cells treated with resveratrol and/or RSL3 (n = 3). (**c**) Quantitation of GPX4, FTH1 and ACSL4 levels in shNC-Caco-2 cells and sh*SIRT3*-Caco-2 cells treated with resveratrol and/or RSL3 (n = 3). (**d**) Representative IF image indicating SIRT3 in shNC-Caco-2 cells and sh*SIRT3*-Caco-2 cells treated with resveratrol and/or RSL3 (n = 3). Scale bars: 200 μm. (**e**) Quantitation of the MFI of SIRT3 in shNC-Caco-2 cells and sh*SIRT3*-Caco-2 cells treated with resveratrol and/or RSL3 (n = 3). The data are presented as the mean ± SD of three independent experiments in triplicates. *P < 0.05, **P < 0.01. Two-way ANOVA (c, d)

The expression of SIRT3 localized to mitochondria in Caco-2 cells was increased after treatment with resveratrol, and SIRT4 and SIRT5 expression did not change significantly. (Figure S6a-c). Caco-2 cells were treated with resveratrol at concentrations of 0, 2.5, 5, 10, 20, and 40 µM for 1 day, 2 days and 3 days, and cell viability was detected by CCK8 assay. We found that Caco-2 cells treated with 20 µM resveratrol for 24 h showed only approximately 10% cell viability (Fig. 7a), so the subsequent in vitro experiments required a concentration of 20 µM resveratrol. The Western blot results suggested that resveratrol increased the expression of GPX4 and FTH1 and decreased the expression of ACSL4 in RSL3-induced shNC-Caco-2 cells but not in sh*SIRT3*-Caco-2 cells (Fig. 7b-c). Similar to the Western blot results, resveratrol decreased the levels of total iron, Fe^2+^, Fe^3+^, MDA and GSSG while increasing the levels of total glutathione and GSH and the GSH/GSSG ratio in shNC-Caco-2 cells but not sh*SIRT3*-Caco-2 cells treated with RSL3 (Figure S6d.). Furthermore, the immunofluorescence results suggested that when SIRT3 was knocked down or cells were treated with RSL3, resveratrol could increase the expression level of SIRT3, but after sh-SIRT3 Caco-2 cells were treated with RSL3, the activation effect of resveratrol on SIRT3 seemed to disappear (Fig. 7d-e).

### Resveratrol reduces ROS by activating the SIRT3/FoxO3a pathway

**Fig. 8:**
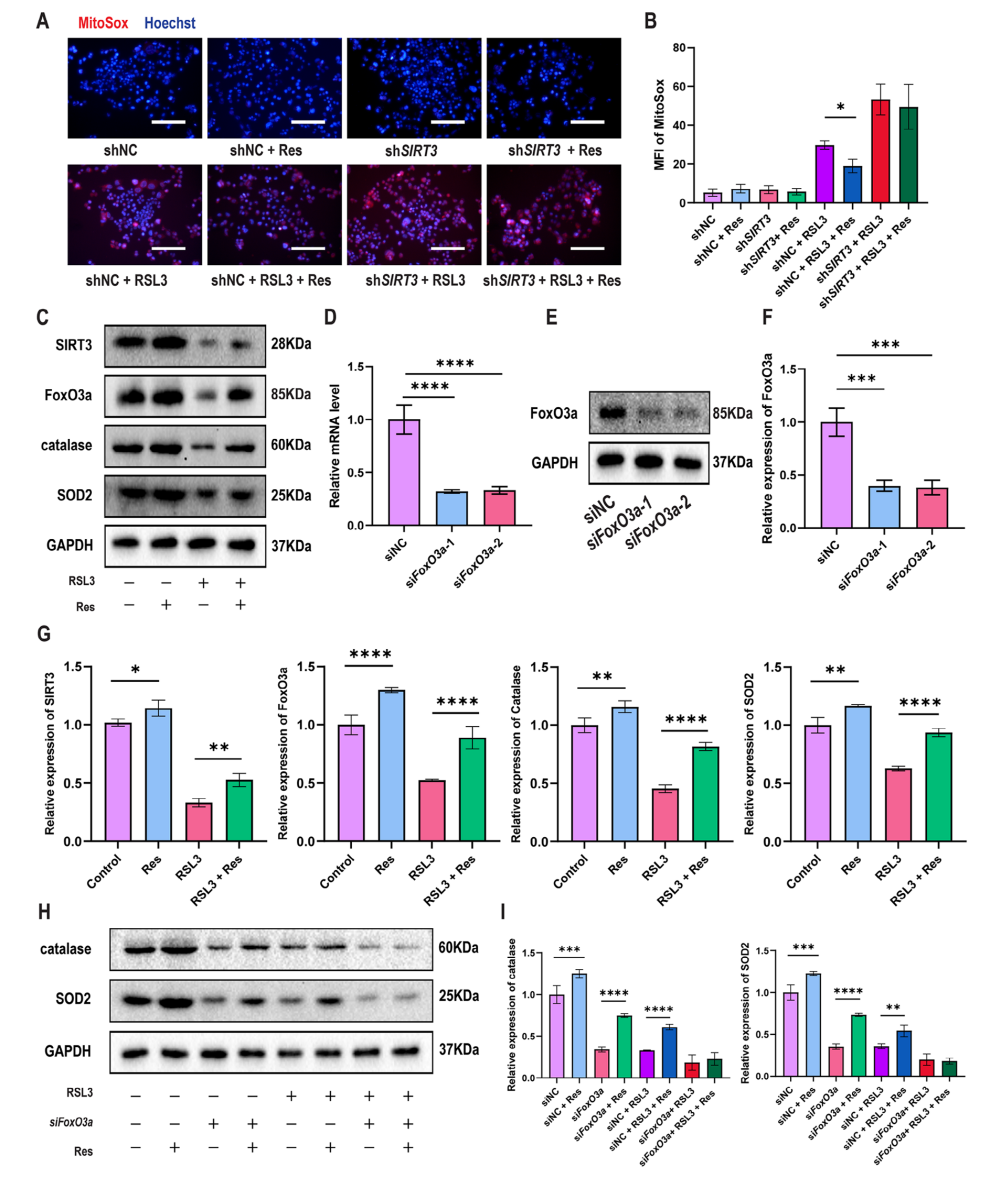
**Resveratrol reduces ROS by activating the SIRT3-FoxO3a pathway**. (**a**) Representative IF image indicating MitoSOX in shNC-Caco-2 cells and sh*SIRT3*-Caco-2 cells treated with resveratrol and/or RSL3 (n = 3). Scale bars: 200 μm. (**b**) Quantitation of the MFI of MitoSOX in shNC-Caco-2 cells and sh*SIRT3*-Caco-2 cells treated with resveratrol and/or RSL3 (n = 3). (**c**) Representative Western blots of SIRT3, FoxO3a, catalase and SOD2 in shNC-Caco-2 cells treated with resveratrol and/or RSL3 (n = 3). (**d**) Quantification of Foxo3a mRNA in siNC-Caco-2 and si*FoxO3a*-Caco-2 cells (n = 3). (**e**) Representative Western blots of FoxO3a in siNC-Caco-2 and si*FoxO3a*-Caco-2 cells (n = 3). (**f**) Quantitation of FoxO3a in siNC-Caco-2 and si*FoxO3a*-Caco-2 cells (n = 3). (**g**) Quantitation of SIRT3, FoxO3a, catalase and SOD2 in shNC-Caco-2 cells treated with resveratrol and/or RSL3 (n = 3). (**h**) Representative Western blots of catalase and SOD2 in siNC-Caco-2 cells and si*FoxO3a*-Caco-2 cells treated with resveratrol and/or RSL3 (n = 3). (**i**) Quantitation of catalase and SOD2 in siNC-Caco-2 and si*FoxO3a*-Caco-2 cells treated with resveratrol and/or RSL3 (n = 3). The data are presented as the mean ± SD of three independent experiments in triplicates. *P < 0.05, **P < 0.01, ***P < 0.001, ****P < 0.0001. One-way ANOVA (d, f), two-way ANOVA (b, g, i)

After MitoSOX Red enters mitochondria, it is rapidly oxidized by ROS and displays red fluorescence after binding to nucleic acids. After treatment with the ferroptosis inducer RSL3, a certain amount of ROS was generated in shNC-Caco-2 cells, and resveratrol successfully ameliorated this trend. A large amount of ROS was produced in RSL3-induced sh*SIRT3*-Caco-2 cells, which could not be ameliorated by resveratrol (Fig. 8a-b). FoxO3a is a transcription factor of SOD2 and catalase and can be actived by SIRT3 (Bai et al. 2023), and the antioxidant genes SOD2 and catalase can reduce the production of ROS (Zhou et al. 2021; Quan et al. 2020). We found that in shNC-Caco-2 cells, whether cells were treated with RSL3 or not, resveratrol could increase FoxO3a, SOD2, and catalase (Fig. 8c and g). FoxO3a was knocked down in Caco-2 cells (Fig. 8d-f), and the expression of SOD2 and catalase decreased. Resveratrol could increase SOD2 and catalase in siFoxo3a-Caco-2 cells, but in RSL3-induced siFoxo3a-Caco-2 cells, resveratrol did not appear to affect the expression of SOD2 and catalase, which could be related to the depletion of Foxo3a caused by the additive effect of siFoxo3a and RSL3 (Fig. 8h-i).

## Discussion

In this study, we first constructed a model of intestinal I/R to simulate the pathophysiological process after acute intestinal obstruction, intussusception, or acute mesenteric artery embolism in humans. Jia et al. confirmed that the longer the ischemia time, the more severe the intestinal I/R injury by fixing the mouse’s intestinal reperfusion time and changing the ischemia time (Jia et al. 2020). Here, we focused on the effect of different reperfusion times on intestinal I/R injury. Referring to the study by Li et al. (Li et al. 2019), we fixed the ischemia time at 60 min and the reperfusion time at 15 min, 30 or 60 min. There was no difference in the Chiu’s score between 30 and 60 min of reperfusion, but in the Western blot results, the expression levels of ZO-1, Occludin, GPX4 and FTH1 were higher after 60 min of reperfusion than after 30 min of reperfusion, similar to the findings of Wang et al. (Wang et al. 2020a). This result suggested that although the expression levels of ZO-1, Occludin and ferroptosis-related proteins GPX4 and FTH1 increased with the extension of reperfusion time, the intestinal mucosa was irreversibly damaged due to intestinal I/R injury.

To simulate intestinal I/R injury in vitro, Caco-2 cells were established according to the study of Liu et al. (Liu et al. 2018). Unlike the intestinal I/R model, H/R did not cause the accumulation of lipid peroxides, changes in the expression of ferroptosis-related proteins or Caco-2 cell death but increased sensitivity to RSL3-induced ferroptosis. This may be because when ischemia occurs in vivo, the regulatory mechanism is more complex, causing not only hypoxia in cells and tissues but also other unknown key molecular changes.

Intestinal I/R injury leads to ferroptosis in intestinal mucosal cells, which was consistent with the study of Li et al. (Li et al. 2019). In this study, the levels of total iron, Fe^2+^, and Fe^3+^ in the blood increased, suggesting that intestinal mucosal cells were damaged by intestinal I/R injury and ferroptosis occurred, and the iron in the cells was released into the blood, which increased the blood iron concentration. The lipids on the cell membrane exist in the form of many phospholipids, and the lipids in the cells are divided into monounsaturated fatty acids and polyunsaturated fatty acids. In the presence of excess Fe^2+^ and ROS, polyunsaturated fatty acids are oxidized to generate excess LOP, causing cell membrane damage and ferroptosis (Stockwell 2022). MDA is the product of LOP (Ayala et al. 2014). After the cell membrane is damaged, MDA is released into the blood, and the level of MDA in the blood increases. Furthermore, once the intestinal tract is damaged by hypoxia or I/R injury, the permeability of intestinal epithelial cells increases, and IFABP and the FITC-marked small molecule dextran (FD4) enter the blood circulation through capillaries (Timmermans et al. 2015; Woting et al. 2018). In this study, the levels of IFABP and FD4 in the blood increased. In addition, intestinal mucosal cells were damaged and ruptured, intestinal endotoxin was released into the blood, and LDH, IL6, and TNF-α levels in the blood increased, resulting in a systemic inflammatory response. In this study, HE results indicated that there was no significant difference in Chiu’s score between 30 and 60 min of reperfusion, while ZO-1, Occludin and SIRT3 increased significantly after 60 min of reperfusion (Fig. 1). This could be because as the reperfusion time was prolonged, the intestinal tissue began to compensate, but the damage to the intestinal mucosa was irreversible. At the same time, we also noticed that there was no difference in the levels of factors such as IL6, LDH, and TNFα in serum at 60 min of reperfusion and 30 min of reperfusion, which might be because systemic compensation had not yet occurred. In the study by Li et al. (Li et al. 2019), the number of ferroptotic mitochondria per field was the highest at 30 min of reperfusion, and the number of ferroptotic mitochondria per field after 240 min of reperfusion showed no difference from that of the control group (Response letter Fig. 1). Therefore, we speculated that with prolonged reperfusion time, the levels of related factors in serum will return to normal levels.

In the absence of hypoxia or I/R injury, normal cells can regulate cell membrane damage, and there are three main regulatory pathways for ferroptosis. The first pathway is the GSH/GPX4 pathway, which involves the cystine/glutamate transporter (Wang et al. 2020b), p53 regulatory axis (Jiang et al. 2015) or glutamine pathway (Gao et al. 2015). The second pathway is related to iron metabolism (Chen et al. 2022; Hou et al. 2016; Sun et al. 2016). The third pathway is related to lipid metabolism (Yang et al. 2016; Dodson et al. 2019; Kagan et al. 2017). In addition, some studies have indicated that the FSP1-CoQ10 pathway plays an important role in ferroptosis (Doll et al. 2019; Bersuker et al. 2019). In our study, the reduced GSH level and GSH/GSSG ratio decreased, and the oxidized GSSG level increased in the process of intestinal I/R. According to research by Quader et al., the GSH/GSSG ratio is greater than 100 in resting cells, but in the presence of ROS, the ratio becomes as low as 10:1 or even 1:1 (Quader et al. 2022). GPX4 is a key enzyme that catalyzes GSH resistance against LPO (Maiorino et al. 2018). In this study, when I/R occurred, the levels of GPX4 and GSH decreased, leading to an increase in LPO production. On the other hand, FTH1 is a part of the ferritin complex and can inhibit ferroptosis in Parkinson’s disease by ferritinophagy (Tian et al. 2020). ACSL4 is a member of the ACSL family that activates polyunsaturated fatty acids, which can be oxidized and trigger ferroptosis (Doll et al. 2017). In this study, FTH1 expression decreased and ACSL4 expression increased when I/R occurred.

Sirtuin3, an NAD^+^-dependent deacetylase, localizes to mitochondria and is involved in almost all aspects of mitochondrial metabolism and homeostasis, protecting mitochondria from various forms of damage (Zhang et al. 2020). SIRT3 accelerates the rate of ROS clearance in mitochondria by enhancing the degree of acetylation of various enzymes (Denu 2017). In this study, we found that SIRT3 expression decreased in the I/R model and RSL3-induced ferroptosis, which was attenuated by resveratrol. FoxO3a is a downstream gene of SIRT3, and FoxO3a loses its activity after ubiquitination, phosphorylation or acetylation (Zhou et al. 2021). SIRT3 can deacetylate FoxO3a to restore its activity, promote the transcription of downstream SOD2 and catalase, increase the expression of SOD2 and catalase, and reduce the generation of ROS (Bai et al. 2023; Quan et al. 2020). After Caco-2 cells were treated with RSL3, we found that the expression level of SIRT3 decreased, and we speculated that more FoxO3a was acetylated. Furthermore, the expression level of FoxO3a was reduced, which resulted in a lower expression level of the active form of FoxO3a.

Resveratrol has been found in at least 72 plant species, such as mulberries, peanuts, and grapes, and is characterized by simple extraction and low cost (Meng et al. 2021). As an agonist of SIRT3, it has antioxidant effects (Zhang et al. 2020; Zhuang et al. 2019). According to the resveratrol instructions, 10 µM resveratrol treatment of MCF7 cells for 60 h only caused 10% MCF-7 cell death. Ulrich et al. demonstrated that 30 µM resveratrol treatment of HCT116 cells for 24 h only caused approximately 10% cell death (Ulrich et al. 2006). In this study, treatment with 20 µM resveratrol for 24 h resulted in only approximately 10% cell death. Furthermore, after treatment with 20% FBS, the cell growth rate was faster, and the cell density in the shNC-Caco-2 group was too high at 48 and 72 h. Therefore, 20 µM resveratrol was used in subsequent experiments to treat Caco-2 cells for 24 h. In this study, resveratrol increased SIRT3 resistance to intestinal I/R injury and ferroptosis, but in the SIRT3 knockout or knockdown group, resveratrol did not ameliorate intestinal I/R injury and ferroptosis. The above experimental results suggest that resveratrol ameliorated the generation of ROS through the Sirt3-FoxO3a pathway, thereby inhibiting the occurrence of ferroptosis.

There are some shortcomings in this study regarding why ferroptosis occurred in the reperfusion stage but not in the reoxygenation stage. This may be related to the body having a more complex regulatory mechanism. Additionally, the study of different ischemia times is not mentioned. Whether the ischemia or reperfusion phase is more damaging to intestinal mucosal tissue may be addressed in future studies.

## Conclusions

To date, this is the first study to show that resveratrol ameliorates intestinal ischemia-reperfusion injury by activating SIRT3 and reducing ferroptosis. Resveratrol can reduce intestinal ischemia-reperfusion injury by activating the SIRT3/FoxO3a pathway, increasing the expression of SOD2 and catalase, reducing ROS and LPO production, compensating for the GSH/GPX4 pathway and inhibiting ferroptosis. Although resveratrol cannot reverse damage to the intestinal mucosa, we suspect that the use of resveratrol will greatly improve patient prognosis.

### Electronic supplementary material

Below is the link to the electronic supplementary material.

Supplementary Material 1

## Data Availability

The data sets used or analyzed during the study are available from the corresponding author upon reasonable request.

## List of Abbreviations

I/R: Ischemia-reperfusion
ROS: Reactive oxygen species
LPO: Lipid peroxidation
SIRT3: Sirtuin 3
FoxO3a: Forkhead box O3
SOD2: Superoxide dismutase 2
GSH: Glutathione
GPX4: Glutathione peroxidase 4
GSSG: Glutathione oxidized
ACSL4: Acyl-coenzyme A synthetase long-chain family member 4
FTH1: Ferritin heavy chain 1
ZO-1: Zonula occludens protein 1
MDA: Malondialdehyde
FD4: Fluorescein isothiocyanate dextran 4
IFABP: Intestinal fatty acid binding protein
LDH: Lactate dehydrogenase

## References

[CR29] Amri A, Chaumeil JC, Sfar S, Charrueau C (2012). Administration of resveratrol: what formulation solutions to bioavailability limitations?. J Control Release.

[CR44] Ayala A, Muñoz MF, Argüelles S. Lipid peroxidation: production, metabolism, and signaling mechanisms of malondialdehyde and 4-hydroxy-2-nonenal. Oxid. Med. Cell. Longev. 2014; 2014: 360438. 10.1155/2014/360438. 10.1155/2014/360438 PMC4066722 24999379

[CR35] Bai J, Pagano RE (1997). Measurement of spontaneous transfer and transbilayer movement of BODIPY-labeled lipids in lipid vesicles. Biochemistry.

[CR26] Bai H (2023). Mitochondria-derived H2O2 triggers liver regeneration via FoxO3a signaling pathway after partial hepatectomy in mice. Cell Death Dis.

[CR28] Baur JA, Sinclair DA (2006). Therapeutic potential of resveratrol: the in vivo evidence. Nat Rev Drug Discov.

[CR57] Bersuker K (2019). The CoQ oxidoreductase FSP1 acts parallel to GPX4 to inhibit ferroptosis. Nature.

[CR38] Bhat AA (2018). Tight Junction Proteins and Signaling Pathways in Cancer and inflammation: a functional crosstalk. Front Physiol.

[CR21] Chen D (2021). iPLA2β-mediated lipid detoxification controls p53-driven ferroptosis independent of GPX4. Nat Commun.

[CR10] Chen X, Comish PB, Tang D, Kang R (2021). Characteristics and biomarkers of ferroptosis. Front Cell Dev Biology.

[CR50] Chen H (2022). Ferroptosis and its multifaceted role in Cancer: Mechanisms and Therapeutic Approach. Antioxid (Basel).

[CR36] Chiu C-J (1970). Intestinal mucosal lesion in Low-Flow States: I. A morphological, hemodynamic, and metabolic reappraisal. Arch Surg.

[CR31] de Sá Coutinho D, Pacheco MT, Frozza RL, Bernardi A. Anti-inflammatory Effects of Resveratrol: mechanistic insights. Int J Mol Sci. 2018;19. 10.3390/ijms19061812. 10.3390/ijms19061812 PMC6032205 29925765

[CR62] Denu RA. SIRT3 Enhances Mesenchymal Stem Cell Longevity and Differentiation. Oxid. Med. Cell. Longev. 2017; 2017: 5841716. 10.1155/2017/5841716. 10.1155/2017/5841716 PMC5499245 28717408

[CR54] Dodson M, Castro-Portuguez R, Zhang DD (2019). NRF2 plays a critical role in mitigating lipid peroxidation and ferroptosis. Redox Biol.

[CR61] Doll S (2017). ACSL4 dictates ferroptosis sensitivity by shaping cellular lipid composition. Nat Chem Biol.

[CR56] Doll S (2019). FSP1 is a glutathione-independent ferroptosis suppressor. Nature.

[CR1] Eltzschig HK, Eckle T (2011). Ischemia and reperfusion–from mechanism to translation. Nat Med.

[CR33] Fox AC (2012). The endogenous bacteria alter gut epithelial apoptosis and decrease mortality following Pseudomonas aeruginosa pneumonia. Shock.

[CR25] Fu B (2017). Resveratrol rescues cadmium-induced mitochondrial injury by enhancing transcriptional regulation of PGC-1α and SOD2 via the Sirt3/FoxO3a pathway in TCMK-1 cells. Biochem Biophys Res Commun.

[CR49] Gao M (2015). Glutaminolysis and transferrin regulate ferroptosis. Mol Cell.

[CR37] Hacioglu A (2005). Protective effect of leptin against ischemia-reperfusion injury in the rat small intestine. BMC Gastroenterol.

[CR51] Hou W (2016). Autophagy promotes ferroptosis by degradation of ferritin. Autophagy.

[CR41] Jia Y (2020). Metformin protects against intestinal ischemia-reperfusion injury and cell pyroptosis via TXNIP-NLRP3-GSDMD pathway. Redox Biol.

[CR48] Jiang L (2015). Ferroptosis as a p53-mediated activity during tumour suppression. Nature.

[CR55] Kagan VE (2017). Oxidized arachidonic and adrenic PEs navigate cells to ferroptosis. Nat Chem Biol.

[CR4] Li Y (2019). Ischemia-induced ACSL4 activation contributes to ferroptosis-mediated tissue injury in intestinal ischemia/reperfusion. Cell Death Differ.

[CR14] Li Y (2020). Inhibitor of apoptosis-stimulating protein of p53 inhibits ferroptosis and alleviates intestinal ischemia/reperfusion-induced acute lung injury. Cell Death Differ.

[CR7] Li J (2020). Ferroptosis: past, present and future. Cell Death Dis.

[CR13] Li X et al. Targeting Ferroptosis: Pathological Mechanism and Treatment of Ischemia-Reperfusion Injury. Oxid. Med. Cell. Longev. 2021; 2021: 1587922. 10.1155/2021/1587922. 10.1155/2021/1587922 PMC8568519 34745412

[CR43] Liu L (2018). Mir-381-3p knockdown improves intestinal epithelial proliferation and barrier function after intestinal ischemia/reperfusion injury by targeting nurr1. Cell Death Dis.

[CR34] Liu J (2020). NOX1/NADPH oxidase in bone marrow-derived cells modulates intestinal barrier function. Free Radic Biol Med.

[CR19] Mahoney-Sánchez L (2021). Ferroptosis and its potential role in the physiopathology of Parkinson’s Disease. Prog Neurobiol.

[CR59] Maiorino M, Conrad M, Ursini F (2018). GPx4, lipid peroxidation, and cell death: discoveries, Rediscoveries, and Open Issues. Antioxid Redox Signal.

[CR5] Matsuo S (2013). Cyclic arginine-glycine-aspartate attenuates acute lung injury in mice after intestinal ischemia/reperfusion. Crit Care.

[CR63] Meng T (2021). Anti-inflammatory action and mechanisms of Resveratrol. Molecules.

[CR17] Mou Y (2019). Ferroptosis, a new form of cell death: opportunities and challenges in cancer. J Hematol Oncol.

[CR27] Pastor RF (2019). Resveratrol, human health and winemaking perspectives. Crit Rev Food Sci Nutr.

[CR58] Quader S, Van Guyse JFR (2022). Bioresponsive Polymers for Nanomedicine-Expectations and reality!. POLYMERS-BASEL.

[CR40] Quan Y et al. Mitochondrial ROS-Modulated mtDNA: A Potential Target for Cardiac Aging. Oxid. Med. Cell. Longev. 2020; 2020: 9423593. 10.1155/2020/9423593. 10.1155/2020/9423593 PMC7139858 32308810

[CR22] Schwer B, Verdin E (2008). Conserved metabolic regulatory functions of sirtuins. Cell Metab.

[CR23] Shen T (2022). Activating SIRT3 in peritoneal mesothelial cells alleviates postsurgical peritoneal adhesion formation by decreasing oxidative stress and inhibiting the NLRP3 inflammasome. Exp Mol Med.

[CR9] Stockwell BR (2022). Ferroptosis turns 10: emerging mechanisms, physiological functions, and therapeutic applications. Cell.

[CR52] Sun X (2016). Activation of the p62-Keap1-NRF2 pathway protects against ferroptosis in hepatocellular carcinoma cells. Hepatology.

[CR60] Tian Y (2020). FTH1 inhibits ferroptosis through Ferritinophagy in the 6-OHDA model of Parkinson’s Disease. Neurotherapeutics.

[CR45] Timmermans K (2015). Circulating iFABP levels as a marker of intestinal damage in trauma patients. Shock.

[CR6] Ucar BI (2020). Effects of endothelin receptor blockade and COX inhibition on intestinal I/R injury in a rat model: experimental research. Int J Surg.

[CR65] Ulrich S (2006). Peroxisome proliferator-activated receptor gamma as a molecular target of resveratrol-induced modulation of polyamine metabolism. Cancer Res.

[CR47] Wang L (2020). ATF3 promotes erastin-induced ferroptosis by suppressing system Xc. Cell Death Differ.

[CR42] Wang Z (2020). SIRT3-mediated deacetylation of PRDX3 alleviates mitochondrial oxidative damage and apoptosis induced by intestinal ischemia/reperfusion injury. Redox Biol.

[CR16] Wang Y (2021). Quercetin alleviates acute kidney injury by inhibiting ferroptosis. J Adv Res.

[CR11] Wang P (2021). Mitochondrial ferritin attenuates cerebral ischaemia/reperfusion injury by inhibiting ferroptosis. Cell Death Dis.

[CR46] Woting A, Blaut M (2018). Small intestinal permeability and gut-transit Time determined with low and high Molecular Weight Fluorescein Isothiocyanate-Dextrans in C3H mice. Nutrients.

[CR20] Wu J (2021). Ferroptosis in liver disease: new insights into disease mechanisms. Cell Death Discovery.

[CR18] Wu X, Li Y, Zhang S, Zhou X (2021). Ferroptosis as a novel therapeutic target for cardiovascular disease. THERANOSTICS.

[CR30] Xia N, Daiber A, Förstermann U, Li H (2017). Antioxidant effects of resveratrol in the cardiovascular system. Br J Pharmacol.

[CR8] Xie Y (2016). Ferroptosis: process and function. Cell Death Differ.

[CR32] Xu S et al. SIRT1/3 Activation by Resveratrol Attenuates Acute Kidney Injury in a Septic Rat Model. Oxid. Med. Cell. Longev. 2016; 2016: 7296092. 10.1155/2016/7296092. 10.1155/2016/7296092 PMC5149703 28003866

[CR2] Yan HF, Tuo QZ, Yin QZ, Lei P (2020). The pathological role of ferroptosis in ischemia/reperfusion-related injury. ZOOLOGICAL Res.

[CR53] Yang WS, Stockwell BR (2016). Ferroptosis: death by lipid peroxidation. Trends Cell Biol.

[CR3] Yellon DM, Hausenloy DJ (2007). Myocardial reperfusion injury. N Engl J Med.

[CR24] Zhang J (2020). Mitochondrial sirtuin 3: New emerging biological function and therapeutic target. THERANOSTICS.

[CR12] Zhao Z (2020). XJB-5-131 inhibited ferroptosis in tubular epithelial cells after ischemia-reperfusion injury. Cell Death Dis.

[CR15] Zhao WK, Zhou Y, Xu TT, Wu Q, Ferroptosis. Opportunities and Challenges in Myocardial Ischemia-Reperfusion Injury. Oxid. Med. Cell. Longev. 2021; 2021: 9929687. 10.1155/2021/9929687. 10.1155/2021/9929687 PMC8557044 34725566

[CR39] Zhou C (2021). SIRT3 alleviates neuropathic pain by deacetylating FoxO3a in the spinal dorsal horn of diabetic model rats. Reg Anesth Pain Med.

[CR64] Zhuang Y et al. Resveratrol Attenuates Oxidative Stress-Induced Intestinal Barrier Injury through PI3K/Akt-Mediated Nrf2 Signaling Pathway. Oxid. Med. Cell. Longev. 2019; 2019: 7591840. 10.1155/2019/7591840. 10.1155/2019/7591840 PMC6915002 31885814

