# Supplementary material for: Resveratrol reduces ROS-induced ferroptosis by activating SIRT3 and compensating the GSH/GPX4 pathway
Source: Mol Med. 2023 Oct 19;29:137. doi: 10.1186/s10020-023-00730-6 (PMC10588250; doi:10.1186/s10020-023-00730-6)

Supplementary Information for

**Resveratrol reduces ROS-induced ferroptosis by activating SIRT3 and compensating the GSH/GPX4 pathway**

**Authors:** Xingjie Wang^1, *^, Tianli Shen^1, *^, Jie Lian^2^, Kai Deng^1^, Chao Qu^1^, Enmeng Li^1^, Gan Li^1^, Yiwei Ren^1^, Zijun Wang^1^, Zhengdong Jiang^1^, Xuejun Sun^1, #,^ Xuqi Li^1, #^

Corresponding to: Xuqi Li (lixuqi@163.com), Xuejun Sun

**This file includes:**

Supplementary Figure. 1 to 6

**Other Supplementary Materials for this manuscript include the following:**

The representative pictures of Western Blots in the supplementary material.

**Figure S1**


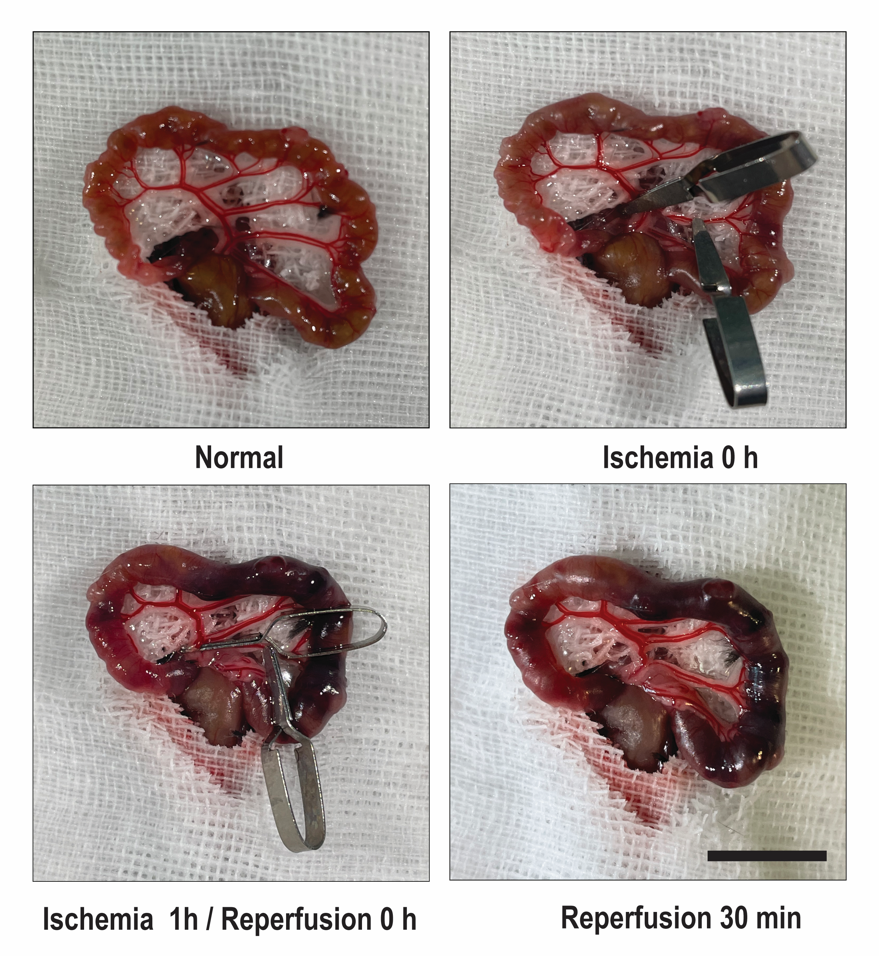


**Figure S1. The method to establish a standardized model of intestinal ischemia-reperfusion in mice.**

Taking 60 minutes of ischemia and 30 minutes of reperfusion as an example. Scale bar: 1cm.

**Figure S2**


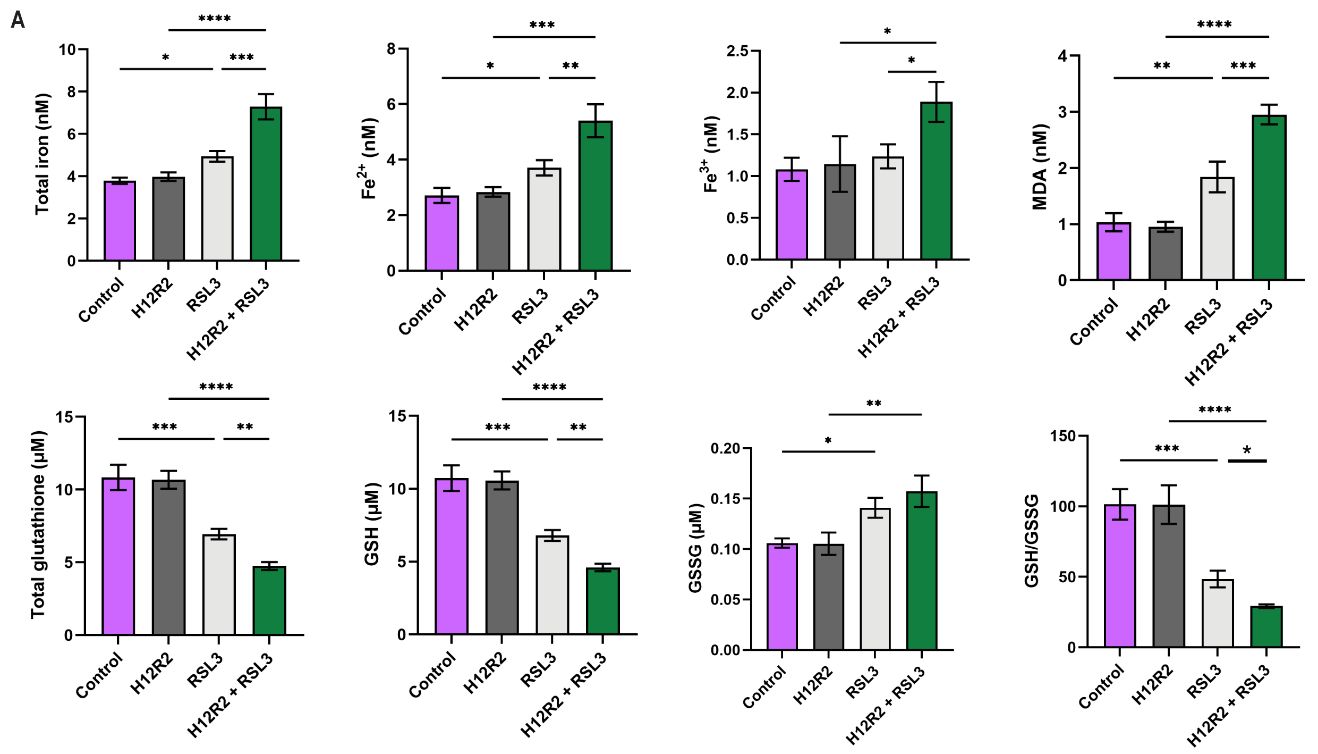


**Figure S2. H/R increases the sensitivity of RSL3 to induce ferroptosis in Caco-2 cells.**

(a) The levels of total iron, Fe^2+^, Fe^3+^, MDA, total glutathione, oxidized glutathione, reduced glutathione levels, and the GSH/GSSG ratio in cells treated with or without H/R and/or RSL3 (n=5). Data are presented as the mean ± SD of three independent experiments in triplicates. **P* < 0.05, ***P*< 0.01, ****P*< 0.001, *****P* < 0.0001. Two-way ANOVA (a).

**Figure S3**


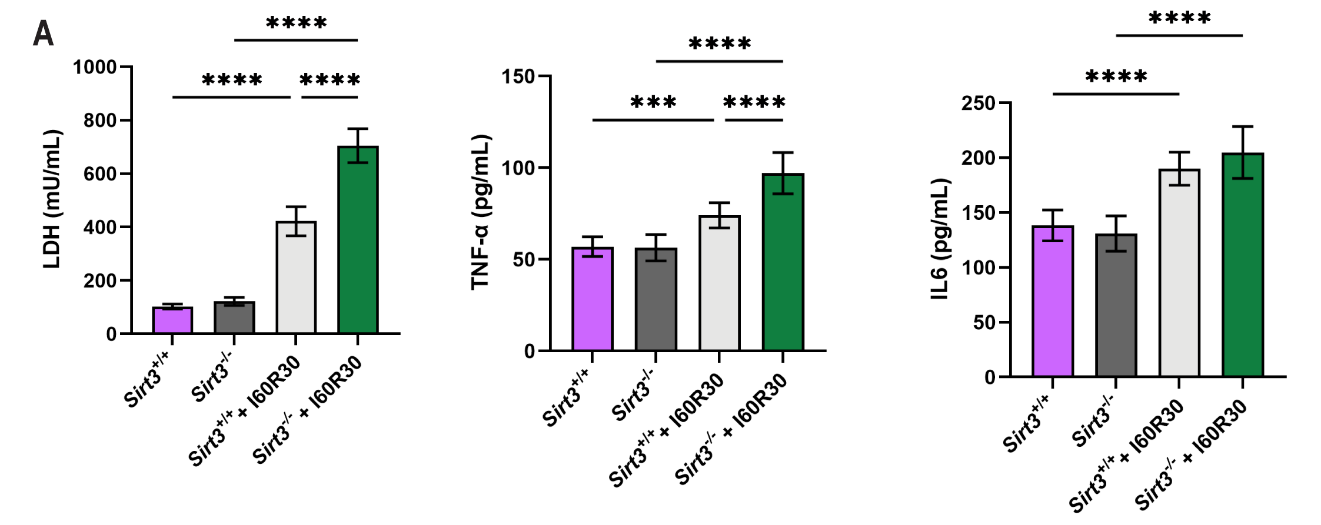


**Figure S3. *Sirt3*^-/-^ increases intestinal I/R injury and ferroptosis in a mouse I/R model.**

(a) Quantification of LDH, TNF-α and IL6 levels in the serum of Sirt3+/+ and Sirt3-/- mice after I/R or without I/R (n=8). Data are presented as the mean ± SD. all experiments were repeated in triplicate. ***P< 0.001, ****P < 0.0001. One-way ANOVA (a).

**Figure S4**


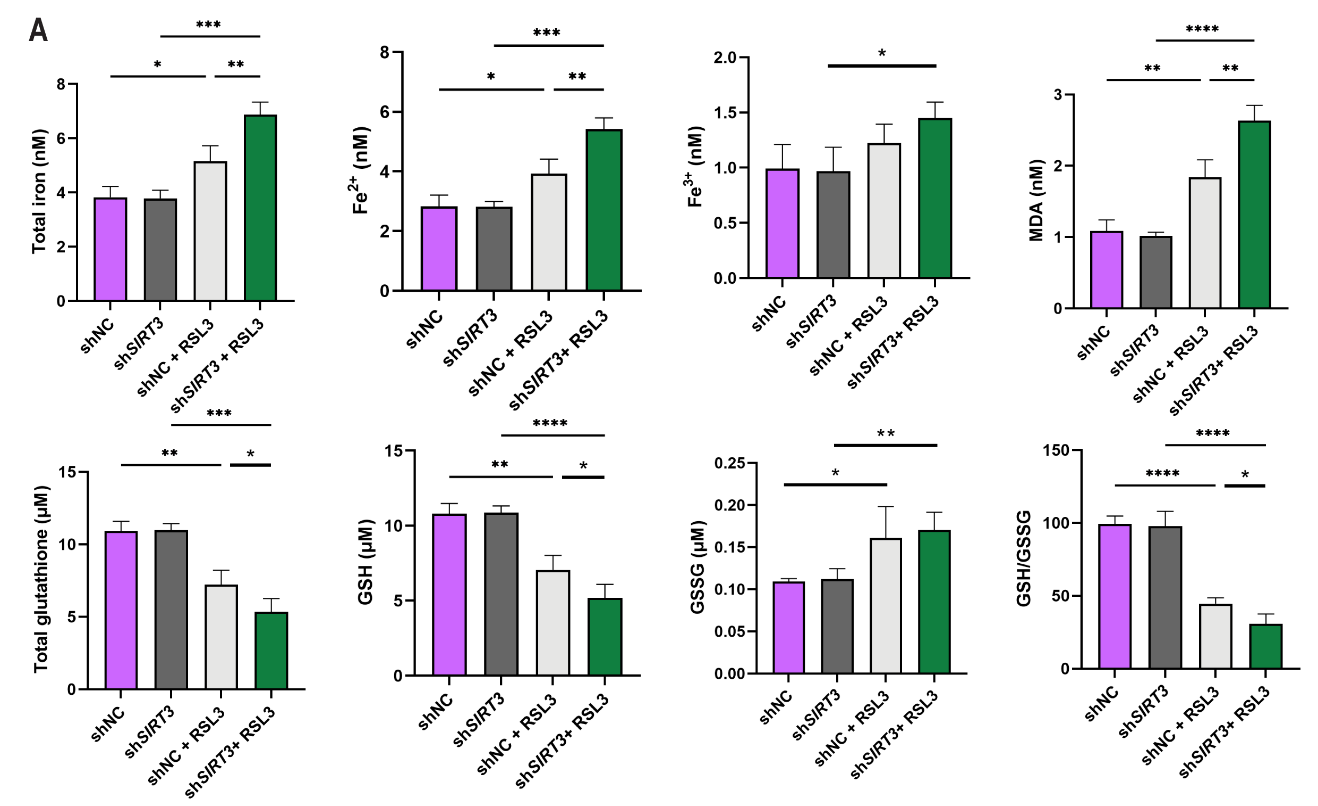


**Figure S4. *SIRT3* knockdown increases sensitivity to RSL3-induced ferroptosis.**

(a) Total iron, Fe2+, Fe3+, MDA, total glutathione, oxidized glutathione, reduced glutathione levels and GSH/GSSG ratio in cells in shNC and sh*SIRT3*-Caco-2 cells treated with or without RSL3 (n=5). Data are presented as the mean ± SD of three independent experiments in triplicates. **P* < 0.05, ***P*< 0.01, ****P*< 0.001, *****P* < 0.0001. Two-way ANOVA (a).

**Figure S5**


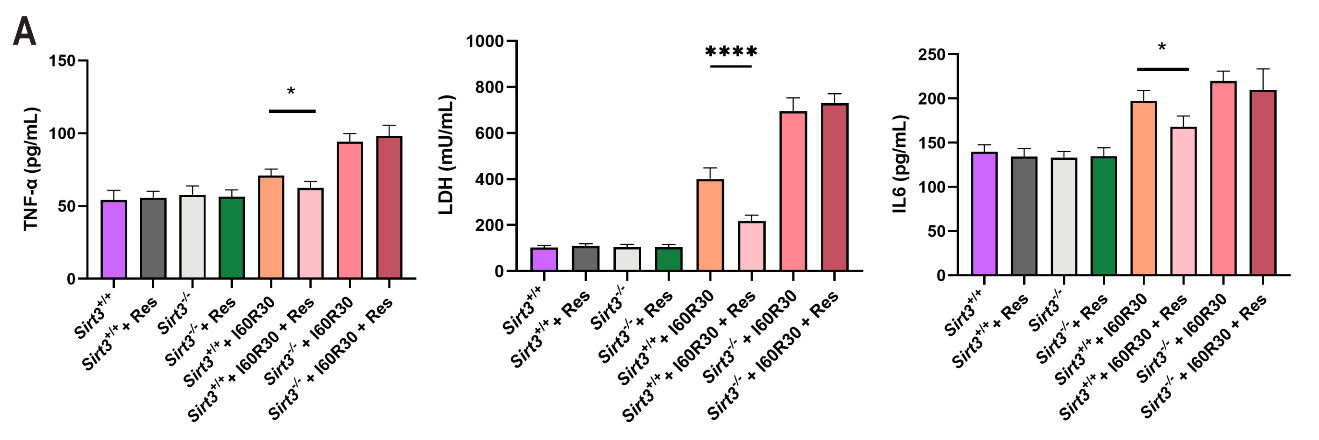


**Figure S5.** **Resveratrol ameliorates intestinal I/R injury in a mouse I/R model.**

(a) The levels of IL6, LDH, and TNF-α in serum from *Sirt3*^+/+^ and *Sirt3*^-/-^ mice treated with resveratrol for 2 weeks and/or I/R (n=8). Data are presented as the mean ± SD. all experiments were repeated in triplicate. **P* < 0.05, *****P* < 0.0001. Two-way ANOVA (a).

**Figure S6**

**
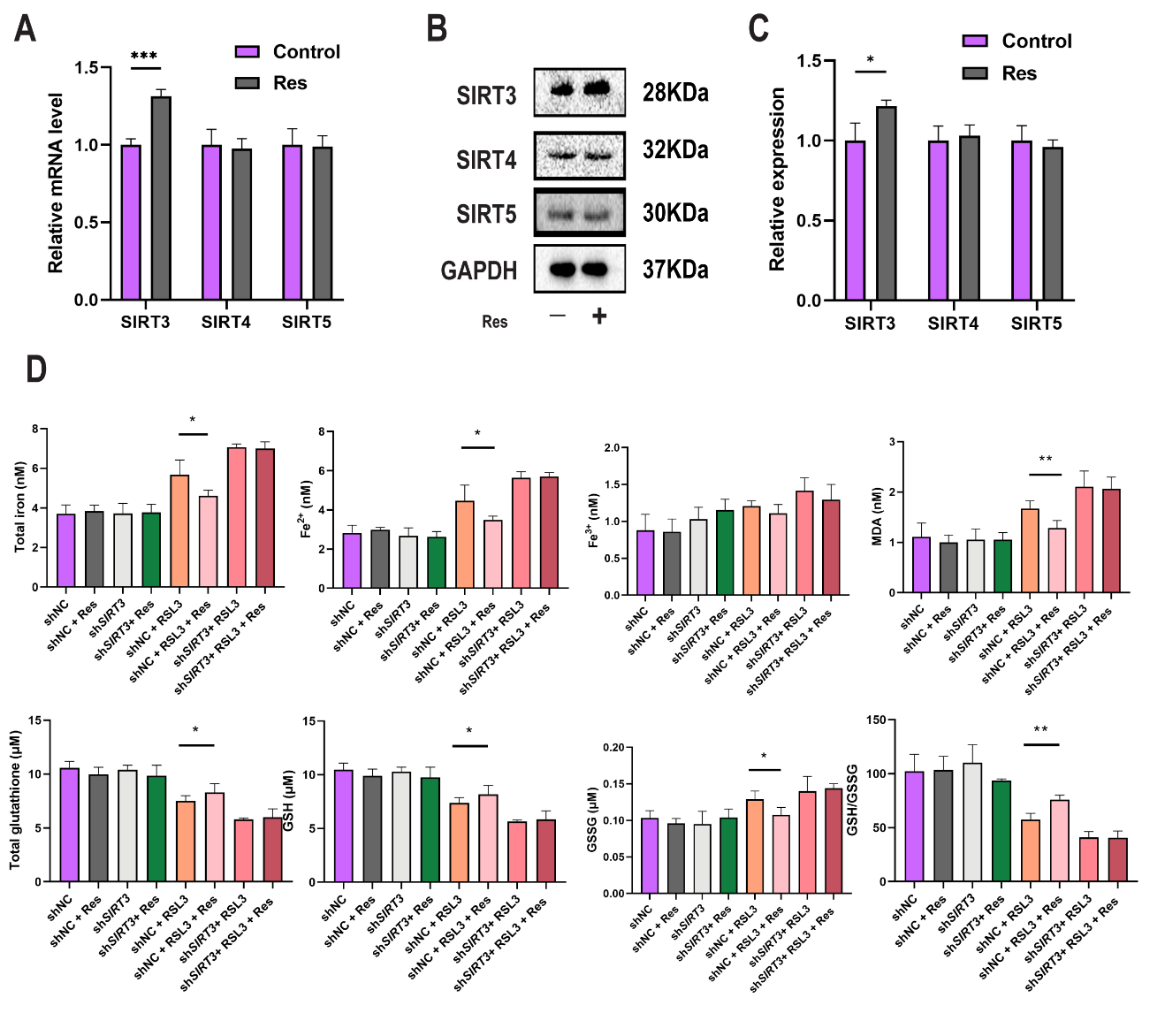
**

**Figure S6. Resveratrol attenuates RSL3-induced ferroptosis dependent on *SIRT3* activation.**

(a) Quantification of *SIRT3, SIRT4 and SIRT5* mRNA in Caco-2 cells treated with or without resveratrol (n=5). (b) Representative Western blots of *SIRT3, SIRT4 and SIRT5* expression in Caco-2 cells treated with or without resveratrol (n=3). (c) Quantification of *SIRT3, SIRT4 and SIRT5* expression in Caco-2 cells treated with or without resveratrol (n=3). (d) The levels of total iron, Fe^2+^, Fe^3+^, MDA, total glutathione, oxidized glutathione, reduced glutathione levels and the ratio of GSH/GSSG in shNC-Caco-2 cells and sh*SIRT3*-Caco-2 cells treated with resveratrol and/or RSL3 (n=5). Data are presented as the mean ± SD of three independent experiments in triplicates. **P* < 0.05, ***P*< 0.01, ****P*< 0.001. Student's t test (a, c), two-way ANOVA (d).

**Other Supplementary Materials**

**Figure 1d**

ZO-1 Occludin


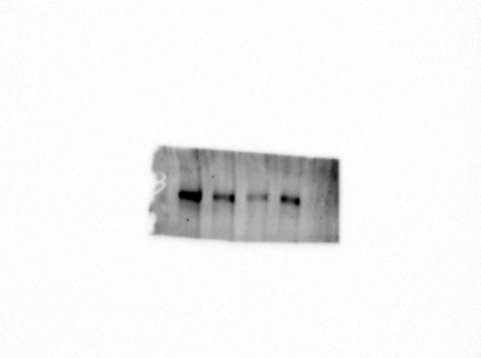




SIRT3 GAPDH


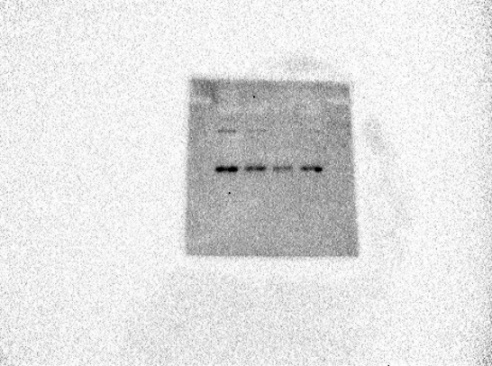

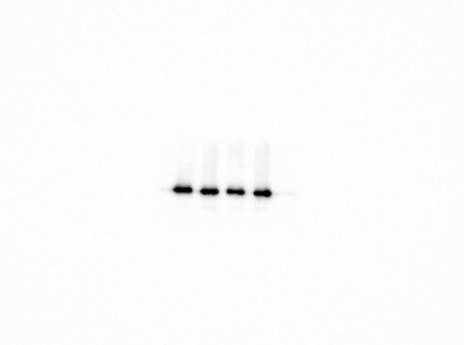


**Figure 2b**

GPX4 FTH1


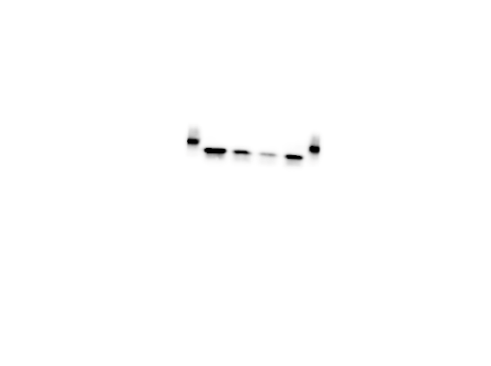

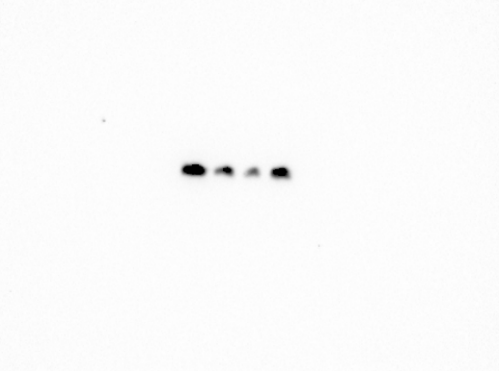


ACSL4 GAPDH


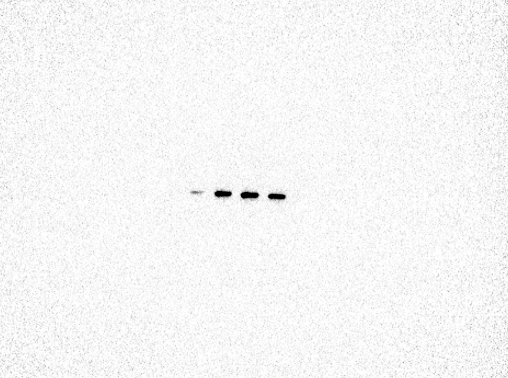

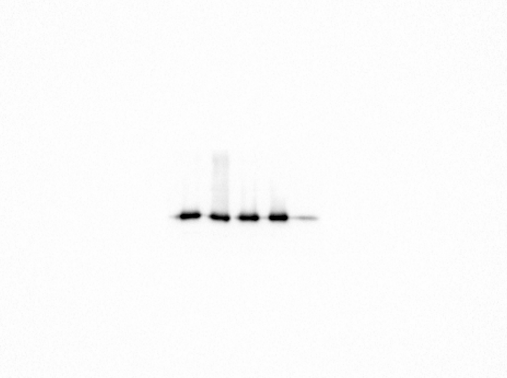


**Figure 3b**

SIRT3 GAPDH


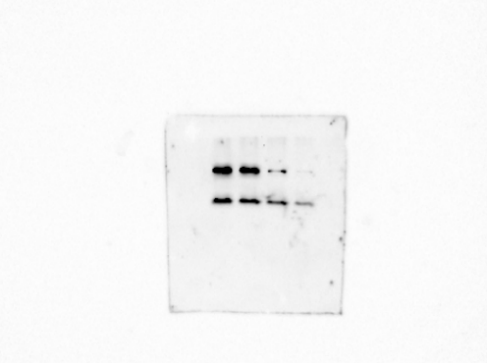

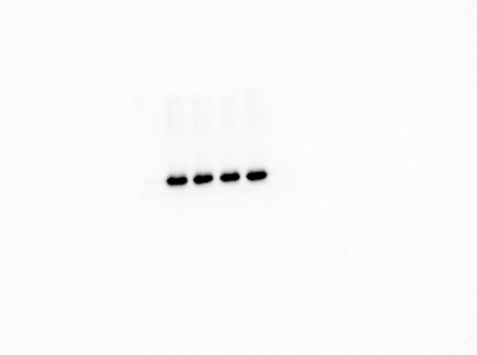


**Figure 4d**

ZO-1 Occludin


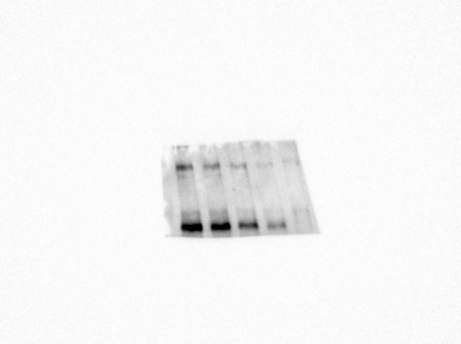

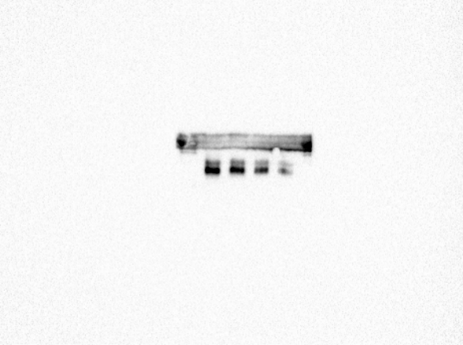


GPX4 FTH1


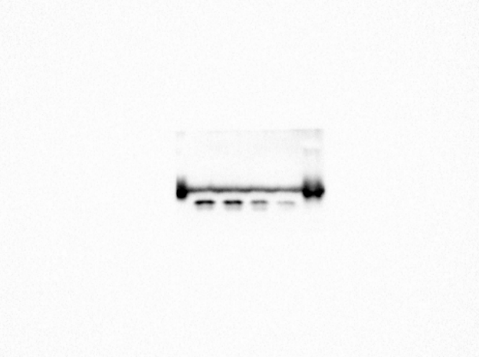

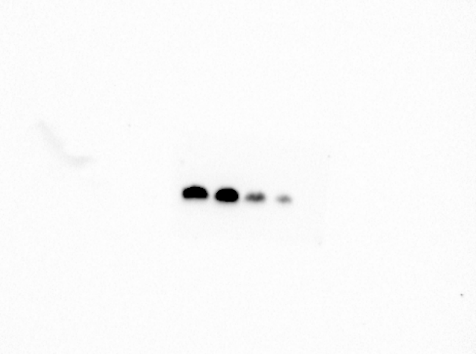


SIRT3 ACSL4


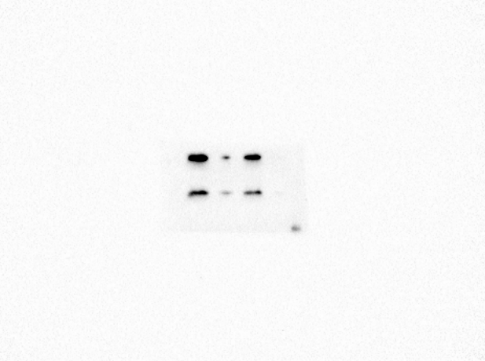

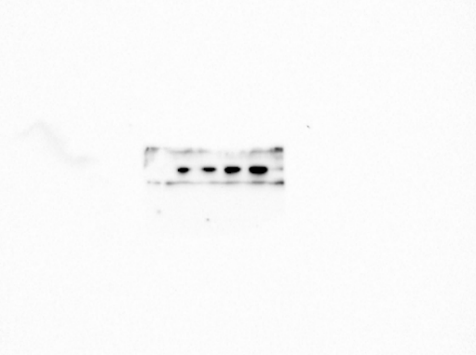


GAPDH


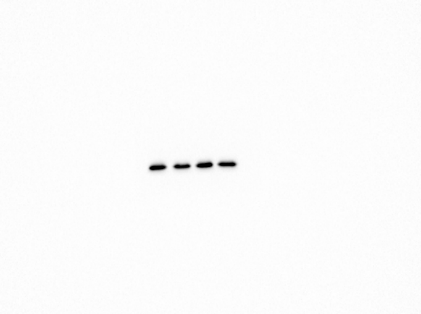


**Figure 5a**

SIRT3 GAPDH


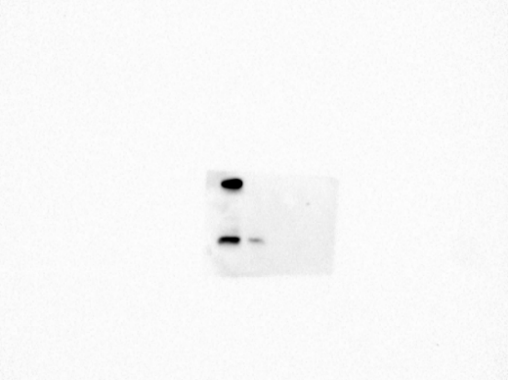

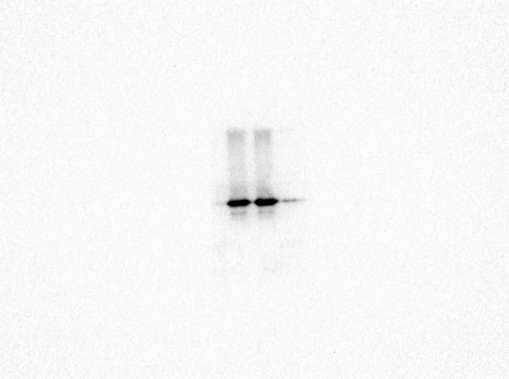


**Figure 6c**

ZO-1 Occludin


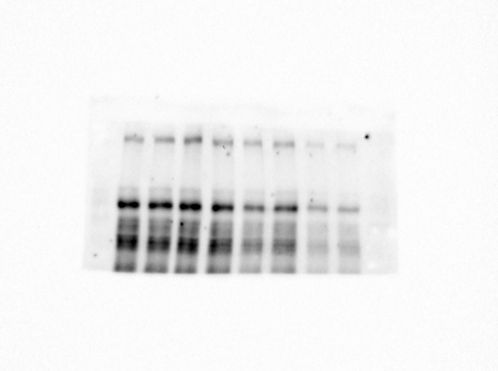

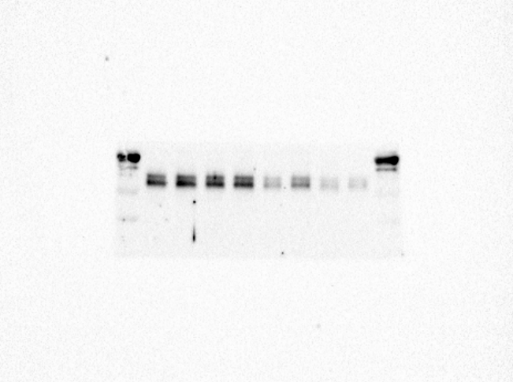


SIRT3 GAPDH


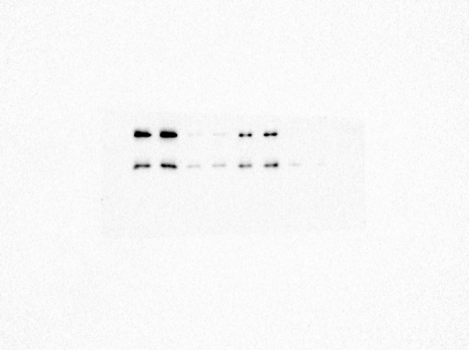

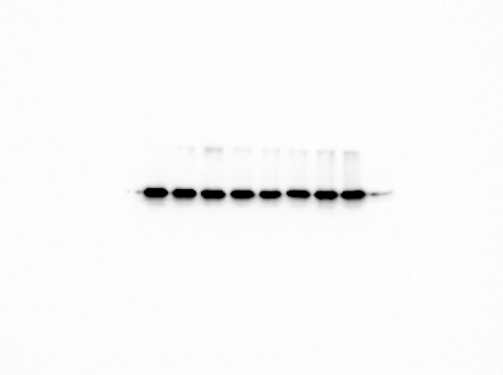


**Figure 6d**

GPX4 FTH1


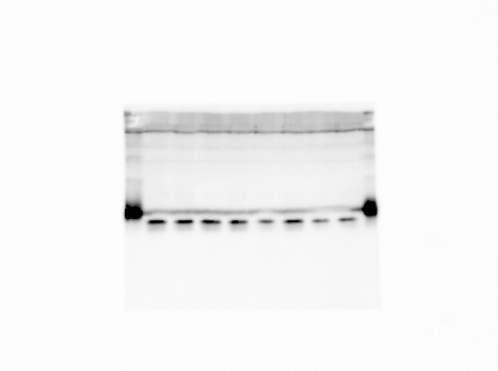

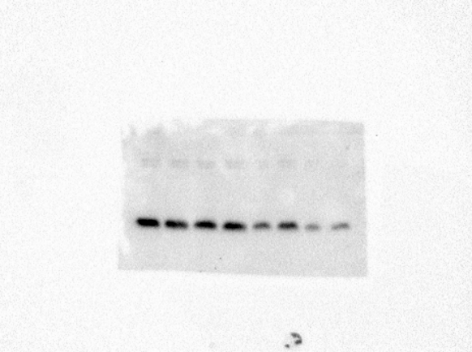


ACSL4 GAPDH


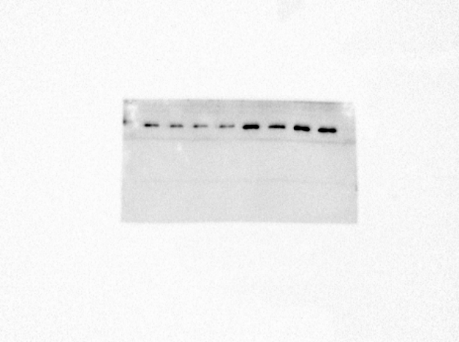

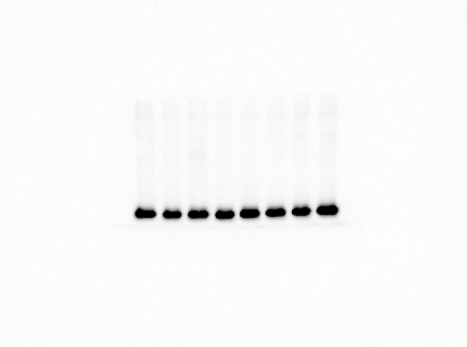


**Figure 7b**

GPX4 FTH1


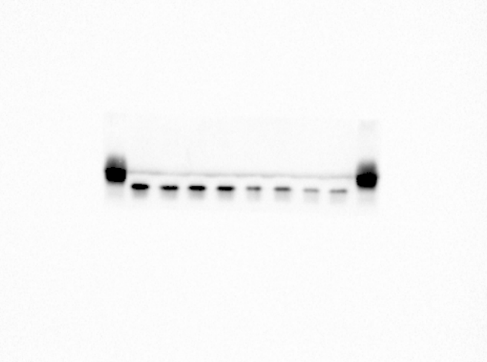

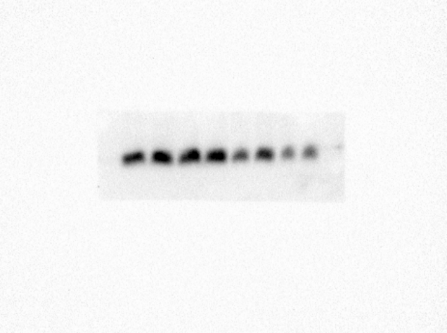


ACSL4 GAPDH


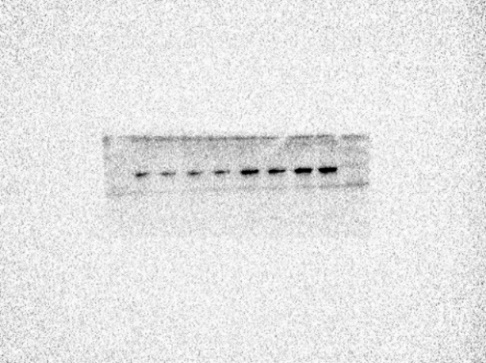

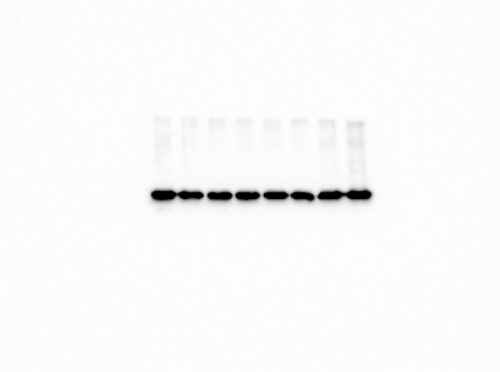


**Figure 8C**

SIRT3 FoxO3a


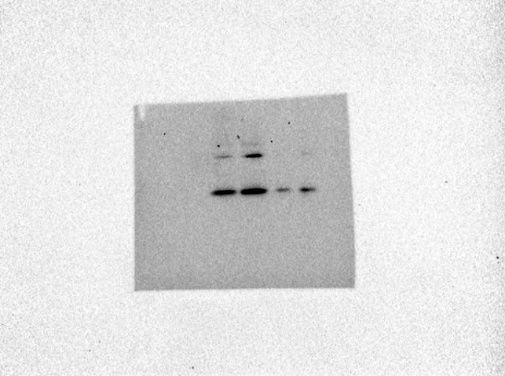

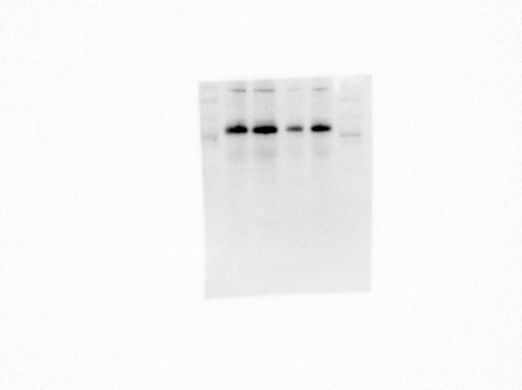


catalase SOD2


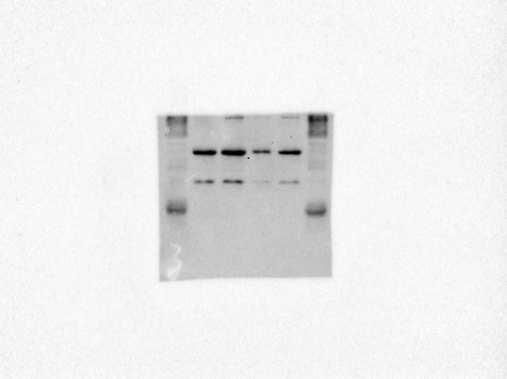

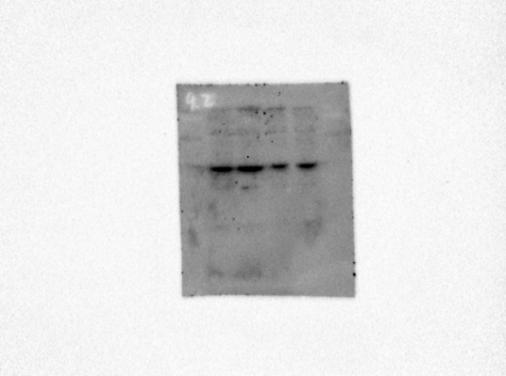


GAPDH


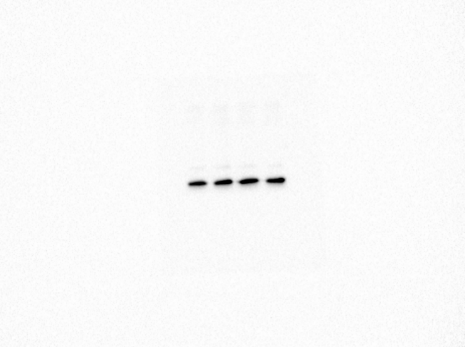


**Figure 8E**

FoxO3a GAPDH


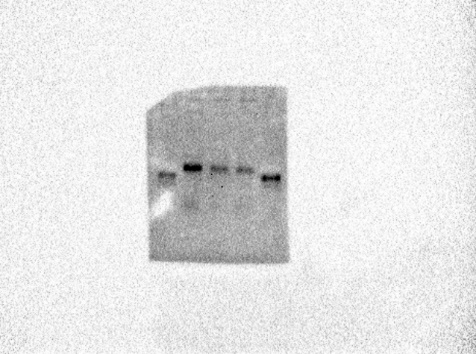

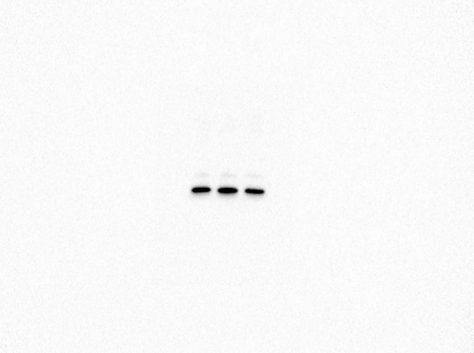


**Figure 8H**

catalase SOD2


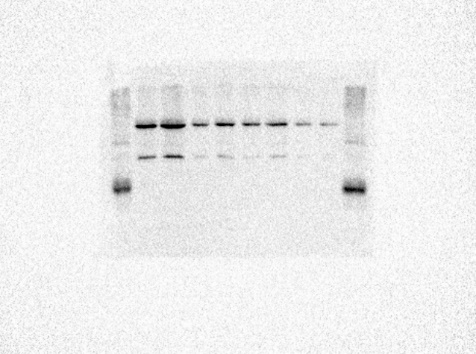

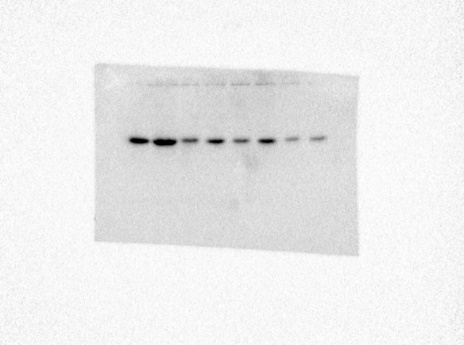


GAPDH


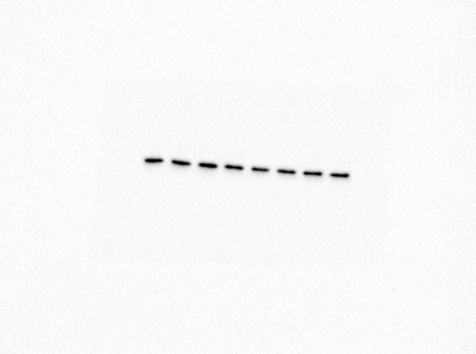


**Figure S6**

SIRT3 SIRT4


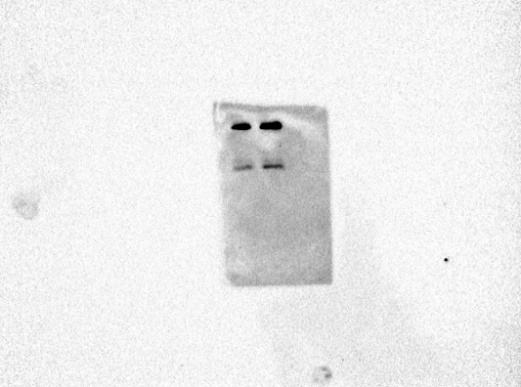

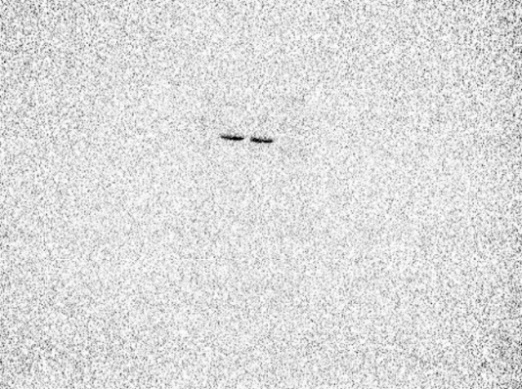


SIRT5 GAPDH


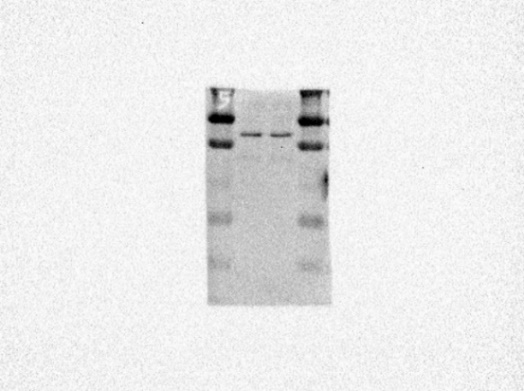

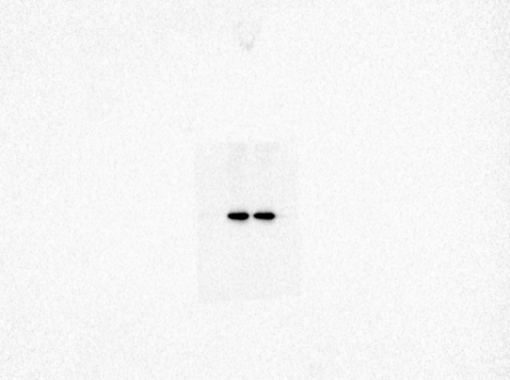

Supplement: Supplementary file 1 — Supplementary Material 1 [file 10020_2023_730_MOESM1_ESM.docx]
